# Potential of Rhizobia Nodulating *Anthyllis vulneraria* L. from Ultramafic Soil as Plant Growth Promoting Bacteria Alleviating Nickel Stress in *Arabidopsis thaliana* L.

**DOI:** 10.3390/ijms231911538

**Published:** 2022-09-29

**Authors:** Marzena Sujkowska-Rybkowska, Anna Rusaczonek, Dorota Kasowska, Krzysztof Gediga, Joanna Banasiewicz, Tomasz Stępkowski, Maciej Jerzy Bernacki

**Affiliations:** 1 Department of Botany, Institute of Biology, Warsaw University of Life Sciences (SGGW-WULS), Nowoursynowska 159, 02-776 Warsaw, Poland; 2 Department of Botany and Plant Ecology, Wrocław University of Environmental and Life Sciences, Grunwaldzki Square 24 A, 50-363 Wroclaw, Poland; 3 Department of Plant Nutrition, Institute of Soil Science, Plant Nutrition and Environmental Protection, Wroclaw University of Environmental and Life Sciences, Grunwaldzka Street 53, 50-357 Wroclaw, Poland; 4 Department of Biochemistry and Microbiology, Institute of Biology, Warsaw University of Life Sciences (SGGW-WULS), Nowoursynowska 159, Building 37, 02-776 Warsaw, Poland; 5 Institute of Technology and Life Sciences, Falenty, Al. Hrabska 3, 05-090 Raszyn, Poland

**Keywords:** antioxidant defense system, Arabidopsis, gene expression, nickel stress, rhizobia, plant growth promotion, ultramafic soil

## Abstract

Rhizobia, which enter into symbiosis with legumes, can also interact with non-legumes and promote plant growth. In this paper, we explored the effects of nickel (Ni, 200 µM) on *Arabidopsis thaliana* (Col-0) inoculated with plant growth-promoting (PGP) rhizobia nodulating ultramafic *Anthyllis vulneraria*. The isolated PGP strains tolerant to Ni were identified as *Rhizobium* sp. and *Bradyrhizobium* sp. The isolates highly differed in their PGP abilities and Ni resistance. Without Ni-stress, the plants inoculated with most isolates grew better and had higher photosynthetic efficiency than non-inoculated controls. Nickel treatment increased Ni concentration in inoculated plants. Plant growth, leaf anatomy, chloroplast ultrastructure, efficiency of photosynthesis, and antioxidant defense system activity were significantly impaired by Ni, however, the majority of these effects were diminished in plants inoculated with the most effective PGP rhizobia. Real-time PCR revealed an increased expression level of genes involved in auxin and gibberellin biosynthesis in the inoculated, Ni-treated plants, and this may have improved shoot and root growth after inoculation with effective isolates. Our results also suggest a positive correlation between Ni-stress parameters and antioxidant defense system activity, and also between the effectiveness of photosynthesis and plant growth parameters. We showed that the selected rhizobia, naturally nodulating Anthyllis on Ni-rich ultramafic soils can promote Arabidopsis growth and increase plant tolerance to Ni by improving different physiological and biochemical mechanisms.

## 1. Introduction

Ultramafic soils are soils naturally rich in nickel, formed as a result of weathering of igneous peridotite and metamorphic serpentinite. These parental rocks have specific mineral composition and the soils derived from them contain elevated levels of potentially toxic metals, especially nickel (Ni), cobalt (Co), and chrome (Cr). Furthermore, ultramafic soils are deficient in nutrients, such as nitrogen (N), potassium (K), phosphorus (P), and calcium (Ca), and have low calcium/magnesium ratio. Consequently, the serpentine syndrome is formed in these soils, which is a specific combination of constraints that exert selective pressure on ultramafic biota [1,2]. Indeed, these soils often support distinctive floras, unique plant communities and soil-born microorganisms that can cope with ultramafic stressful conditions [3,4,5,6,7,8,9,10]. In dry grasslands on ultramafic soils in Poland, representatives of *Fabaceae* (legumes) are abundant and frequent elements of flora both on natural ultramafic outcrops and ultramafic mine soils [11]. The metal-tolerant plants such as *Anthyllis vulneraria* L. (*Fabaceae*), which spontaneously colonize ultramafic soils, must adapt to the constant metal stress and nutrient deficiency. A symbiosis between legumes and nitrogen-fixing bacteria, named ‘rhizobia’, is a major reason why legumes can colonize nutrient-deficient soils and marginal lands contaminated with metals [12,13,14,15]. The legume-rhizobia symbiotic interactions lead to the formation of nodules that are essential organs providing microanaerobic conditions allowing differentiated rhizobia (enclosed in symbiosomes) to fix atmospheric nitrogen and convert it into ammonia used by the host plant [16,17,18]. Our studies on legume-rhizobia symbiosis on ultramafic tailings indicate the presence of rhizobia not only effective in biological nitrogen fixation but also showing the ability to promote plant growth as plant growth promoting bacteria (PGPB) [14]. Apart from their interactions with legumes, rhizobia are known to form growth promoting associations with non-legumes, including monocots such as maize (*Zea mays*), wheat (*Triticum aestivum*), rice (*Oryza sativa*), and dicots such as radish (*Raphanus* *raphanistrum*), carrots (*Daucus carota*), tomato (*Lycopersicon esculentum*), and *Arabidopsis thaliana* [19,20,21,22,23,24,25,26]. The mechanisms of these associations are not clear. Plant growth promoting rhizobia can secrete metabolites that stimulate plant growth and play important roles in environmental stress tolerance [13,16,17,18,19,20,21,22,23,24,25,26,27,28,29,30,31,32]. Application of PGP rhizobia increase plant survival in the presence of hazardous levels of metals mainly by counteracting biomass decrease by improved mineral nutrition and secretion of phytohormones, enhance the production of plant growth regulators (auxins, gibberellins, cytokinins), and stimulate photosynthesis and antioxidant activity [13,16,17,18,19,20,21,22,23,24,25,26,27,28,29,30,31,32]. Moreover, rhizobia also support plant adaptation to metal-polluted sites through chelation of the metals in the soils [14,29,33]. It has been shown that ultramafic soil bacteria are tolerant to nickel [33,34], and they exhibit species-unique cellular response mechanisms under stress conditions [29,34]. The exopolysaccharides and siderophores secreted by rhizobia immobilize toxic ions and help maintain ionic balance in the rhizosphere [29,32,33,34]. Moreover, the use of rhizobia in combination with plants is expected to minimize stress reactions in plants colonizing metal-polluted soils and provide high efficiency for phytoremediation (the use of plants to extract and remove pollutants) [29,32,33,34,35,36,37,38].

Nickel is an essential micronutrient for plant growth and development at low doses, but it becomes toxic at high concentrations [35,36,37,38]. In non-tolerant plants, an excess of Ni (>10 mg kg^−1^) impairs a large variety of processes, and negatively affects the physiological, morphological and biochemical characteristics of plants [35,36,37,38]. Nickel stress inhibits the enzymatic activity, reduces carbohydrate accumulation and chlorophyll biosynthesis, which decreases the efficiency of photosynthesis and causes leaf chlorosis, finally leading to inhibition of plant growth [38,39]. Moreover, excessive amounts of Ni induce oxidative stress and cell death by generating high amount of reactive oxygen species (ROS) produced in multiple cellular organelles. ROS cause oxidative damage of membrane lipids, proteins, and nucleic acids [40,41,42,43,44,45,46,47]. Some of them, such as superoxide radical (O^−^), hydrogen peroxide (H_2_O_2_), hydroxyl radical (OH^•^), and singlet oxygen (^1^O_2_) play a dual role in plant physiology. They not only act as signaling molecules under stressful conditions but are also unavoidable by-products of various biochemical pathways, including photosynthesis and plant-microbes interactions [46,47,48].

Plants colonizing soils contaminated with toxic metals develop mechanisms that enable them to tolerate these conditions. These mechanisms generally do not inhibit metal absorption but provide their internal detoxification. Two basic strategies of the plant response are suggested, excluders (retain metals in their roots) and accumulators (store metal ions in aerial tissues) [49,50]. One of the mechanisms of metal-tolerant plants for coping with Ni stress is to activate the production and accumulation of antioxidants. The non-enzymatic antioxidants (i.e., phenolics, flavonoids, and glutathione), as well as the enzymatic ones [i.e., superoxide dismutase (SOD), catalase (CAT), peroxidase (POD), ascorbate peroxidase (APX), and glutathione reductase (GR)] mitigate the Ni-induced oxidative stress [47,48,49,50,51]. The application of plant growth-promoting bacteria (PGPB) into metal-contaminated soil is another strategy for improving plant tolerance to metal stress. Some studies indicate that rhizobia may alleviate this stress and allow plants to grow on metal-polluted soils [29,49,52,53,54,55].

Taking into account the above data, and the fact that ultramafic dry grasslands are also covered with non-legume plants accompanying Anthyllis, such as *Arabidopsis thaliana* (*Brassicaceae*) [53], in this study we investigated whether inoculation with selected rhizobia, naturally nodulating *Anthyllis vulneraria* on Ni-rich ultramafic soils, can reduce the negative effects of Ni stress by influencing the morphological and physiological characteristics of model *A. thaliana* (L.) plants.

## 2. Results

### 2.1. Rhizobia Identification, Characterization, and Screening for PGP Traits

Seventy bacterial isolates nodulating Anthyllis on ultramafic soils were subjected to PCR-BOX1AR fingerprinting, resulting in their division into several genomic types (Supplementary Figure S1A). For selected isolates representing different BOX-PCR profiles the *glnII* gene was amplified (Supplementary Figure S1B) and selected strains AS5, AS8, AS16, AS52, and AS55 were checked for nodulation ability during authentication test on Anthyllis plants in sterile ultramafic soil. The *glnII* gene was chosen due to a limited intrageneric resolution of the 16S rRNA sequencing approach, particularly evident within rhizobial strains [54]. The results of sequence analysis of the *glnII* genes and alignment with databases deposited in NCBI GenBank revealed that the isolates represented the genera *Rhizobium* (isolates AS5, AS8, AS16, AS52) and *Bradyrhizobium* (isolate AS55) (see Table 1). We failed to sequence the AS14 strain, so it was not considered in further study. For a clear view of the isolate groupings with reference type strains, the phylogeny of *Rhizobium* and *Bradyrhizobium* was constructed (Supplementary Figure S2). The strains AS5 and AS8 (representing one haplotype) exhibited the highest similarity (95.38%) to *Rhizobium tibeticum* strain HAMBI 3177 and *R. tibeticum* type strain CCBAU 85039^T^, although they could potentially represent a new lineage within the *Rhizobium* genus (see Supplementary Figure S2). The isolates AS16 and AS52 carried *glnII* sequences >98% similar, respectively, to *Rhizobium* sp. RMCC TR2021 (100%) and *Rhizobium* sp. strains GG5/GG20/GG37 (98.76%). The isolate AS16 aligned also in one clade with *Rhizobium galegae* type strain HAMBI 540^T^ and *Rhizobium vignae* type strain CCBAU 83006^T^ in the *glnII* phylogeny, while the isolate AS52 with *Rhizobium lusitanum* type strain P1–7^T^ (Supplementary Figure S2). One of the strains, isolate AS55, was most similar to *Bradyrhizobium* sp. strain 7Cha14Z with 99.04% similarity and clustered close to *Bradyrhizobium viridifuturi* type strain SEMIA 690^T^ in the *glnII* tree (Supplementary Figure S2).

Additionally, all tested strains (AS5, AS8, AS16, AS52, and AS55) were confirmed as rhizobia using the spread plate technique with selective rhizobia for the Yeast Extract Congo Red Agar medium (Table 2, Supplementary Figure S3). Morphological characterization of individual colonies showed white bacterial cells after 3–7 days of growth at 28 °C. All tested isolates were Gram-negative and rod-shaped (Table 2, Supplementary Figure S3). Furthermore, all tested strains were positive for ammonia, siderophore, and indole acetic acid (IAA) (1.1 µg mL^−1^ for AS16 to 2.1 µg mL^−1^ for AS55) synthesis, phosphorus solubilization, and ACC deaminase activity (0.32 for 0.93 µmol α-ketobutyrate µg^−1^ protein h^−1^) (Table 2, Supplementary Figure S3).

**Figure 1:**
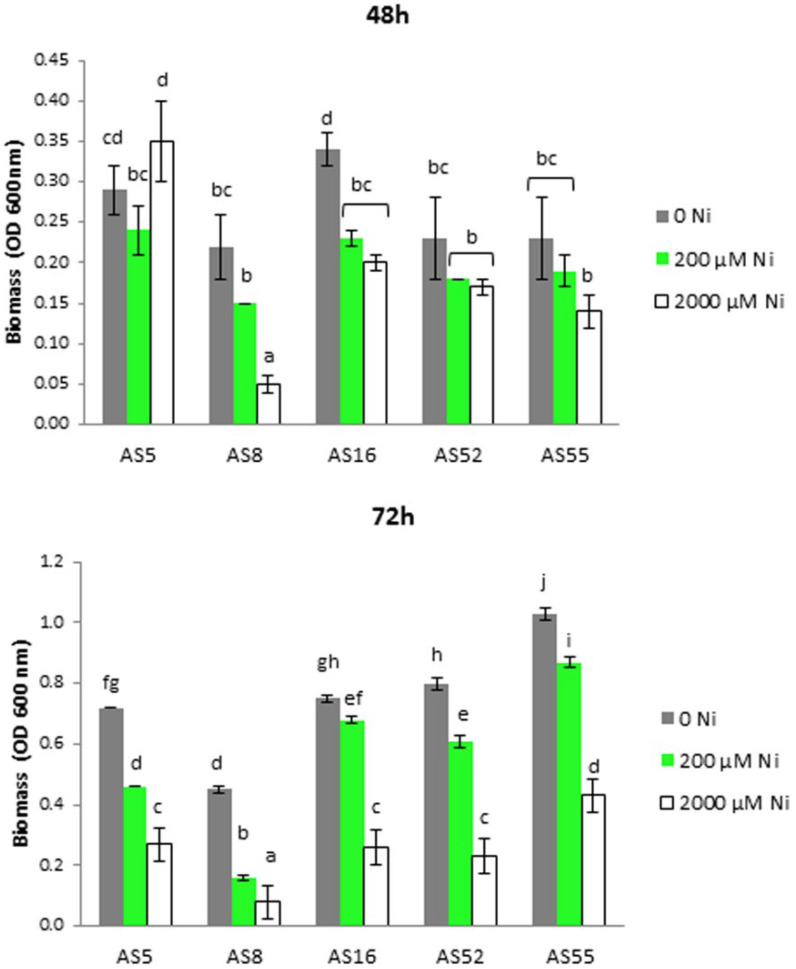
Growth in terms of absorbance at 600 nm (optical density at 600 nm, OD 600) *A. vulneraria* rhizobial isolates supplemented with different concentrations of Ni. Different letters indicate a statistically significant difference among treatments in ANOVA comparisons and the post-hoc Tukey HSD at *p* = 0.05. Error bars represent ± SD.

All tested isolates showed tolerance to the presence of 200 µM Ni according to the experiment on SLP medium (Figure 1). After 48 h, significant growth was observed for the AS5 strain at high Ni concentration (2000 µM). After 72 h, a significant decrease in growth was observed for all strains exposed to the Ni dose, with the smallest inhibition for AS55. The AS8 strain showed the weakest growth in the medium highly contaminated with Ni. Since the presence of 200 µM Ni was tolerated by all rhizobial strains, this concentration was used in further analyses of the potential promoting effect of these bacteria on Arabidopsis growth.

### 2.2. Effect of Rhizobial Strains on Arabidopsis Growth under Normal versus Nickel Stress Conditions

**Figure 2:**
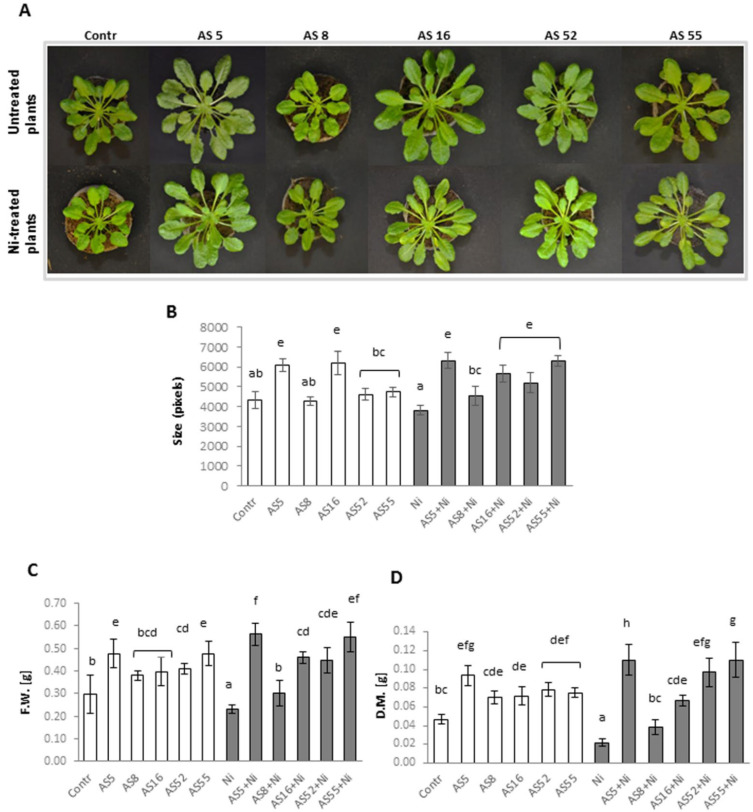
Arabidopsis growth non-exposed (white bars) and exposed to 200 µM Ni (grey bars) for 4 weeks, in the absence or presence of rhizobia strains. (**A**) Pictures of plants cultivated under laboratory non-stress conditions (top row) and exposed to Ni (bottom row), (**B**) rosettes size, (**C**) fresh weigh (FW), and (**D**) dry mass (DM) of Arabidopsis plants. Values are mean ± SD. The same letters indicate the homogenous groups according to the Tukey HSD test at the level *p* = 0.05.

The isolates AS5, AS8, AS16, AS52, and AS55 were evaluated for in vivo PGP potential in the presence of Ni (0 or 200 µM) in Arabidopsis plants. Figure 2 shows the effect of inoculation on the rosette size, fresh weight, and dry mass of Arabidopsis. We found that inoculation with AS5 and AS16 strains significantly promoted rosette growth under control conditions (Figure 2A,B). For the remaining strains, there were no differences in comparison with the non-inoculated control. Moreover, AS5 and AS55 induced a significant gain of fresh weight and AS5, AS16, AS52, and AS55 induced a dry weight increase in Arabidopsis plants as compared with the non-inoculated control (Figure 2D).

Further, the use of nickel at 200 µM resulted in a decrease in the rosette size as well as the fresh weight and dry mass of non-inoculated plants as compared with the untreated control, and the results were significant for the last two parameters (Figure 2A–D). In Ni-treated plants, inoculation with AS5, AS16, AS52, and AS55 strains significantly increased the size of the rosettes and fresh and dry weight of Arabidopsis (Figure 2A–D). The AS8 strain showed the weakest effect on these parameters. The AS5, AS52, and AS55 isolates exhibited the strongest PGP potential in the presence of Ni (Figure 2A–D).

### 2.3. Effect of Rhizobial Inoculants on Root Growth and Lateral Root Formation under Normal versus Nickel Stress Conditions

**Figure 3:**
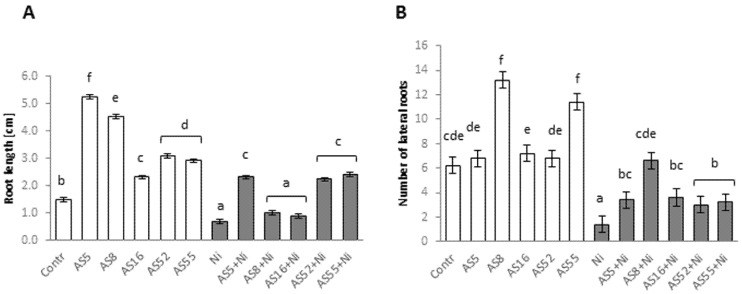
(**A**) Root length and (**B**) number of lateral roots of Arabidopsis plants non-exposed (bars in white) and exposed (bars in grey) to 200µM Ni for 4 weeks in the absence or presence of rhizobia. Error bars represent ± SD. The same letters indicate the homogenous groups according to the Tukey HSD test at the level *p* = 0.05.

Root growth of Arabidopsis under control conditions was significantly promoted by all rhizobial strains (Figure 3A), while AS8 and AS55 ones mainly stimulated the lateral root formation (Figure 3B). Ni inhibited the root growth and the formation of lateral roots of control plants (Figure 3A,B). Inoculation with AS5, AS52, and AS55 strains alleviated the negative effect of Ni on the root length. The lateral root formation was promoted by all rhizobial strains under Ni-stress, especially by the AS8 strain (Figure 3B).

### 2.4. Effect of Rhizobial Inoculants on Gene Expression Level in Arabidopsis under Normal versus Nickel Stress Conditions

**Figure 4:**
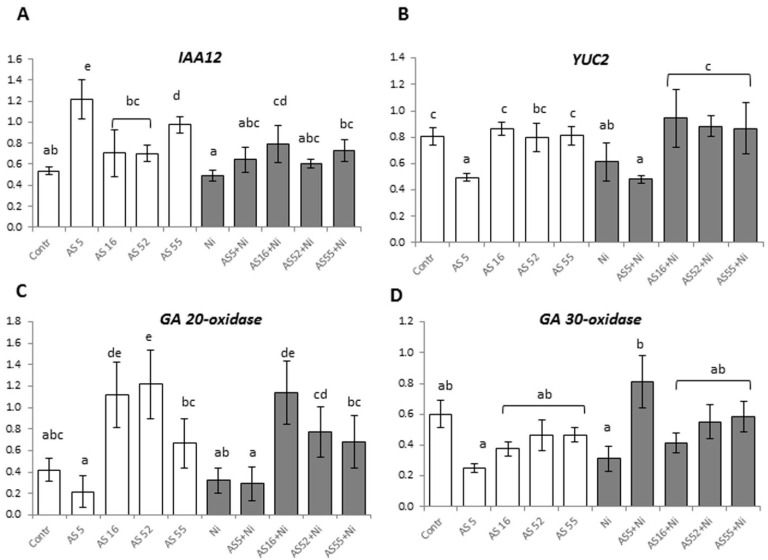
Comparison of real-time RT-PCR expression levels of the genes involved in auxin signaling (*IAA12*) (**A**) and biosynthesis (*YUC2*) (**B**), and gibberellin *GA 20-oxidase* (**C**) and *GA 30-oxidase* (**D**) biosynthesis. Each column represents the expression level (mean and SD) for each gene in each group. Plants non- exposed (bars in white) and exposed to 200 µM Ni (bars in grey) for 4 weeks. Different letters indicate a statistically significant difference among treatments in ANOVA comparisons and the post-hoc Tukey HSD at the level *p* = 0.05. Error bars represent ± SD.

Plant hormones such as auxin and gibberellin regulate various processes of plant growth and development. As the AS8 strain did not promote Arabidopsis growth, it was not included in PCR analysis of the expression of genes connected with auxin and gibberellin biosynthesis. Real-time PCR revealed an increased expression level of the gene involved in auxin signaling (*IAA12*) in control Arabidopsis plants inoculated with AS5 and AS55 strains (Figure 4A). However, the expression of the *YUC2* gene involved in auxin biosynthesis decreased after AS5 application, while other rhizobial strains did not affect the level of its expression (Figure 4B). Moreover, we found higher expression levels of the gene (*GA 20-oxidase*) encoding proteins involved in gibberellin biosynthesis in the leaves of control plants inoculated with AS16, AS52, and AS55 strains (Figure 4C). Bacterial inoculation had no effect on the expression level of *GA30-oxidase* (Figure 4D).

In Ni-treated plants, *IAA12* expression increased after inoculation with AS16 and AS55 strains. *YUC2* expression level was higher after AS16, AS52, and AS55 application in Ni-treated plants. Gene expression involved in gibberellin synthesis was significantly higher in the plants inoculated with AS16 and AS52 for *GA 20-oxidase*, while AS5 inoculation induced *GA 30-oxidase* expression under Ni-stress (Figure 4A–D).

### 2.5. Effect of Rhizobial Strains on Nickel Concentration and Uptake under Normal versus Nickel Stress Conditions

**Figure 5:**
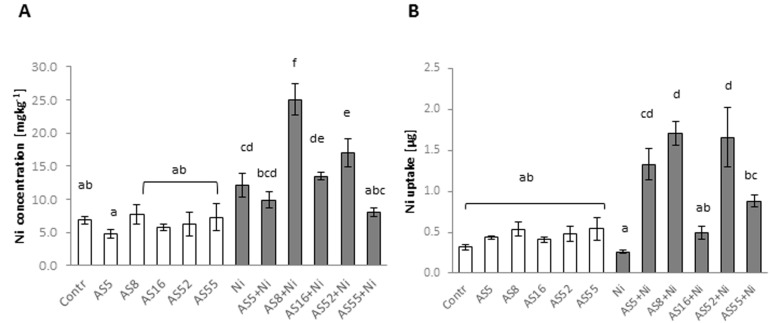
Nickel concentration (**A**) and uptake (**B**) in aboveground parts of Arabidopsis non-exposed (bars in white) and exposed to 200 µM Ni (bars in grey) in the absence or presence of rhizobia strains. Error bars represent ± SD. The same letters indicate the homogenous groups according to the Tukey HSD test at the level *p* = 0.05.

The inoculation with bacterial strains had no effects on Ni concentration in Arabidopsis shoots or Ni uptake in the plants not treated with the metal (Figure 5A,B). The use of Ni resulted in a significant increase in Ni concentration in non-inoculated control plants, without any effect on Ni uptake. The plants inoculated with AS8 and AS52 isolates accumulated the highest levels of Ni. The inoculation with other strains had no significant effect on Ni concentrations (Figure 5A,B). Nickel uptake was the highest in the plants inoculated with AS5, AS8, and AS52 strains and in control plants, it was significantly higher for all rhizobial isolates. In the presence of the AS16 isolate, the same level of Ni uptake was seen as in Ni-treated controls (Figure 5B).

### 2.6. Effect of Rhizobial Inoculants on Leaf Functional Anatomical Traits under Normal versus Nickel-Stress Conditions

**Figure 6:**
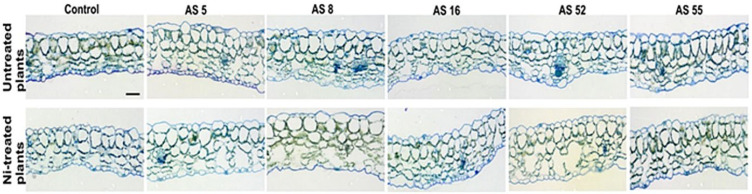
Cross sections of leaves of Arabidopsis non-exposed and exposed to 200 µM Ni, grown under absence or presence of rhizobia. Scale = 50 µm.

Arabidopsis leaves showed a typical dorsiventral structure with mesophyll made of one layer of palisade cells and three layers of spongy parenchyma with large intercellular spaces. Stomata were rare on the adaxial surface and mainly present on the abaxial epidermis (Figure 6, Table 3). Rhizobia inoculation changed the anatomical organization of Arabidopsis leaves under control and Ni-stress conditions mainly by significant leaf thickening (Table 3). Leaf thickening in non-treated plants was associated with the formation of longer mesophyll cells. The plants inoculated with the AS5 and AS16 isolates had significantly thicker leaves than those inoculated with other isolates and non-inoculated control. The bacteria inoculation enlarged the mesophyll cells, the size of air chambers between the spongy mesophyll cells, and the number of spongy cell layers, which was especially significant for AS5- and AS52-inoculated plants (Figure 6, Table 3). Inoculation increased also stomata frequency in both epidermal surfaces as compared with the non-inoculated control (Table 3).

Ni treatment decreased leaf thickness and disturbed mesophyll organization (Figure 6, Table 3). The spongy parenchyma formed larger intercellular spaces than in the non-treated leaves, especially after the bacteria application (Figure 6). Moreover, thinner leaves of Ni-treated non-inoculated plants formed smaller mesophyll cells. These Ni-induced effects were significantly reduced after rhizobia inoculation (Table 3). The plants inoculated and exposed to Ni showed longer palisade cells and more loose spongy parenchyma due to wider air chambers than the non-inoculated plants (Figure 6). Furthermore, Ni-stress caused a significant increase in stomata frequency in the abaxial epidermis in all nickel treatments versus the non-metal control (Table 3).

### 2.7. Effect of Rhizobial Inoculants on Chloroplast Ultrastructure and Photosynthetic Pigment Content under Normal versus Nickel-Stress Conditions

**Figure 7:**
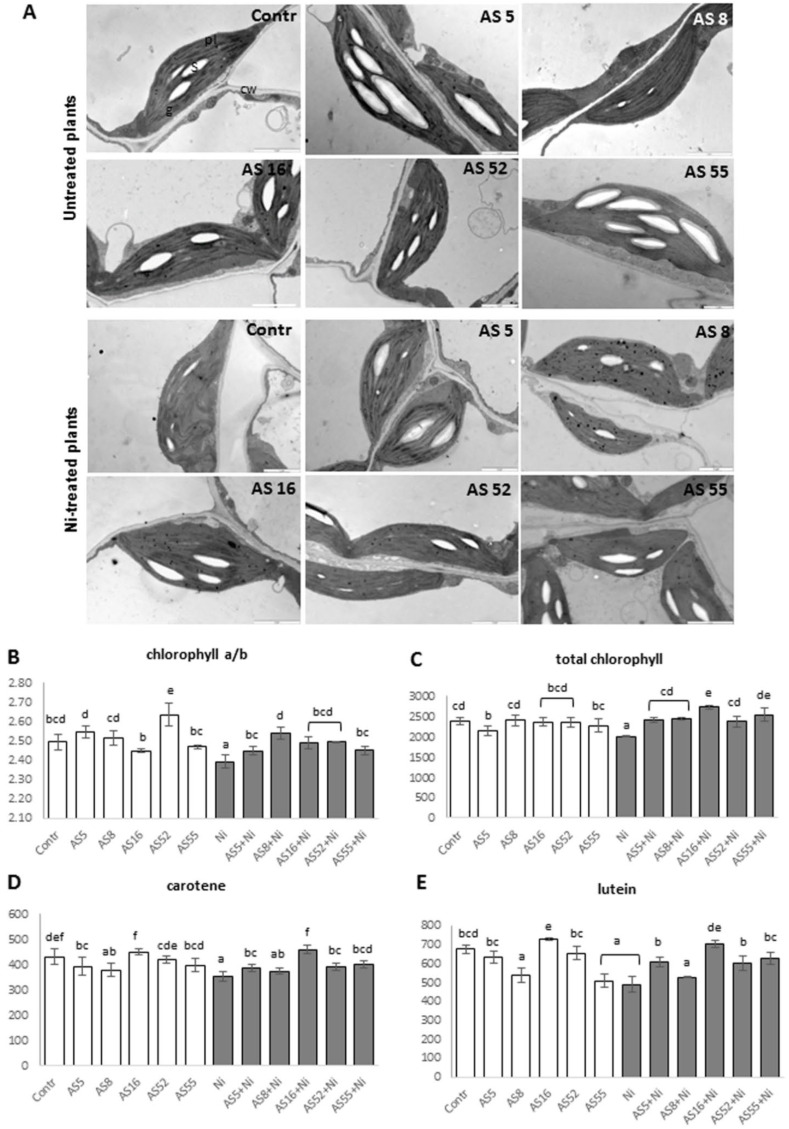
(**A**) Transmission electron micrographs showing leaf chloroplast ultrastructure of Arabidopsis untreated and Ni-treated, grown in the absence (Contr) or presence of rhizobia (AS5, AS8, AS16, AS52, or AS55, respectively). Note that there was higher starch accumulation in the chloroplasts of Arabidopsis leaves inoculated with isolates AS5, AS16, AS52, and AS55 under control and Ni-stress conditions. Abbreviations: cw, cell wall; s, starch grain; g, granum; pl, plastoglobuli. Scale = 2 µm. (**B**) Photosynthetic pigments: chl a/b, (**C**) total chlorophyll (a + b), (**D**) carotene, and (**E**) lutein contents of 4-week-old Arabidopsis plants non-exposed (bars in white) and exposed to 200 µM Ni (bars in grey), in the absence or presence of rhizobia. Different letters indicate a statistically significant difference among treatments in ANOVA comparisons and the post-hoc Tukey HSD at the level *p* = 0.05. Error bars represent ± SD.

Transmission electron microscopy (TEM) showed typical, crescent-like shapes with strongly electron-dense stroma and regular structure of thylakoids in chloroplasts in the mesophyll of non-inoculated plants. The number of starch grains varied between 1 to 3 per chloroplast, and only a few small plastoglobules per chloroplast were observed (Figure 7A). Chloroplasts in the leaves of plants inoculated with AS5, AS52, and AS55 strains had dense stroma with a well-developed thylakoid system and abundantly formed grana with numerous starch grains and small plastoglobules. The chloroplasts of AS8-inoculated plants showed the lowest starch accumulation.

Chloroplasts of Ni-treated non-inoculated plants showed alterations in their shape and inner structure. Numerous chloroplasts were round, with electrontranslucent stroma, and/or thylakoid-free stroma areas with bigger plastoglobules than the non-treated controls. Moreover, the chloroplasts accumulated a small number of starch grains and their internal membranes were often swollen (Figure 7A). These chloroplast deformations were absent in the rhizobia-inoculated plants. In the mesophyll of AS5-, AS52-, and AS55-inoculated plants, chloroplasts looked similar to those from untreated leaves and harbored numerous starch grains, suggesting active photosynthesis despite Ni-stress. Additionally, rhizobia inoculation induced the formation of greater amounts of small plastoglobules under Ni-stress, which was especially noticeable for the AS8 strain (Figure 7A).

Under non-metal conditions, the plants inoculated with the AS52 strain had the highest chl a/b ratio, while total chlorophyll content was similar in the leaves of all inoculated plants (Figure 7B,C). For AS16, we observed significantly higher carotene and lutein content than for all other isolates (Figure 7D,E), while the AS8 strain reduced the content of both compounds in comparison with other isolates and the control (Figure 7D,E).

Ni application reduced the content of all pigments and the chlorophyll a/b ratio in non-inoculated plants (Figure 7B–E). After inoculation, the increase in total chlorophyll and chl a/b ratio was noticed for all strains in comparison with Ni-treated controls. This same result was observed for lutein and carotene content but with the exception of AS8 isolate (Figure 7B–E).

### 2.8. Effect of Rhizobial Inoculants on Chlorophyll a Fluorescence Uptake under Normal versus Nickel-Stress Conditions

**Figure 8:**
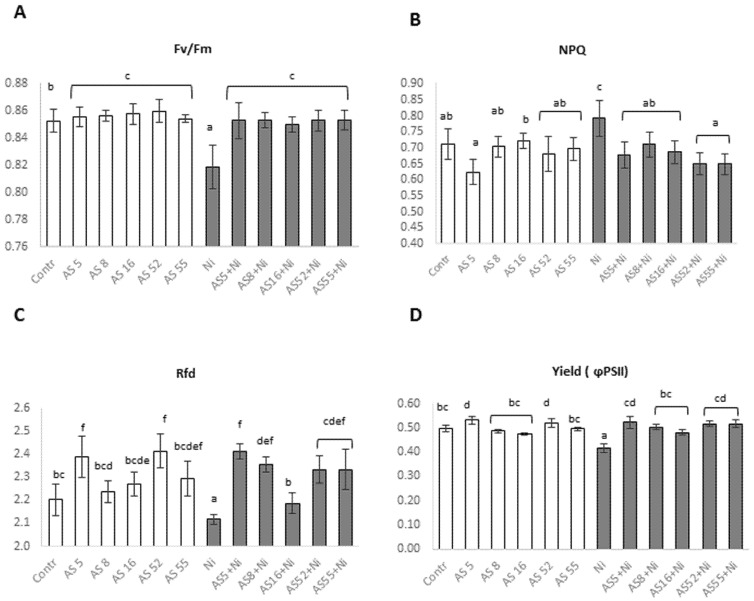
Chlorophyll *a* fluorescence parameters: (**A**) Fv/Fm—maximum efficiency, (**B**) NPQ—non-photochemical quenching, (**C**) Rfd—vitality index, (**D**) ϕPSII—quantum yield of PSII of 4-week-old Arabidopsis plants non-exposed (bars in white) and exposed to 200 µM Ni (bars in grey), in the absence or presence of rhizobia. Error bars represent ± SD. The same letters indicate the homogenous groups according to the Tukey HSD test at the level *p* = 0.05.

In non-metal conditions, the Fv/Fm levels increased following the rhizobia application but we found no significant differences in Fv/Fm value between the rhizobial isolates (Figure 8A). The plant vitality index (Rfd) and the steady-state quantum yield of PSII photochemistry (Yield = ϕPSII) were the highest for AS5- and AS52-inoculated plants (Figure 8C,D). Furthermore, for the AS5 isolate, non-photochemical quenching (NPQ) values were the lowest but the differences between the controls and all the isolates were not significant (Figure 8B).

Ni treatment significantly decreased Fv/Fm, Rfd, and ϕPSII values, whereas the NPQ levels significantly increased in non-inoculated plants (Figure 8A–D). After inoculation, we observed an increase in Fv/Fm for all rhizobia treatments (Figure 8A). The plants inoculated with AS5, AS52, and AS55 strains had the highest Rfd and ϕPSII parameters (Figure 8C,D). In the case of the NPQ, the values decreased after the bacterial inoculation (Figure 8B).

### 2.9. Effect of Rhizobial Inoculants on Reactive Oxygen Species (ROS) Accumulation and Cell Death under Normal versus Nickel-Stress Conditions

**Figure 9:**
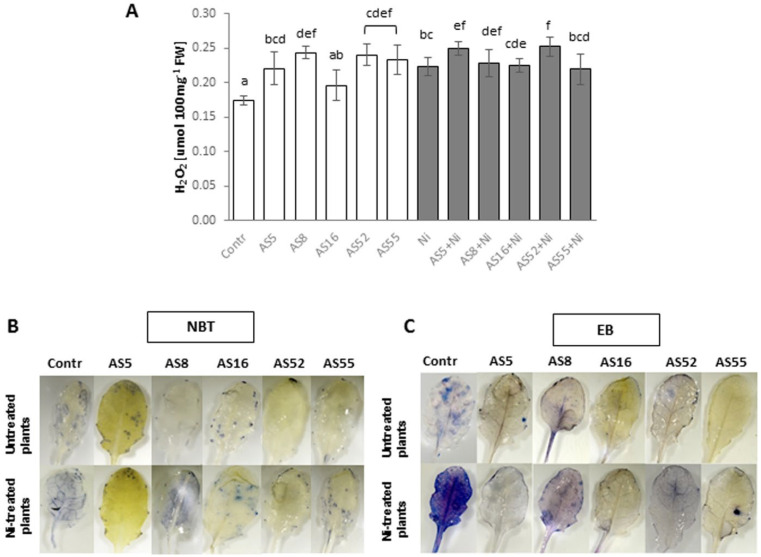
ROS determination and cell death detection in 4-week-old Arabidopsis plants, non-exposed (white bars) and exposed to 200µM Ni (grey bars), in the absence or presence of rhizobia. (**A**) H_2_O_2_, hydrogen peroxide content; (**B**) Visualization of superoxide anion accumulation after NBT staining; (**C**) Cell death visualization after Evans Blue (EB) staining (blue color indicates sites of necrosis). Error bars represent ± SD. The same letters indicate the homogenous groups according to the Tukey HSD test at the level *p* = 0.05.

Spectrophotometric measurement of H_2_O_2_ content in control Arabidopsis leaves revealed that rhizobia application (except for AS16 strain) significantly increased its accumulation (Figure 9A). High levels of H_2_O_2_ observed in plants inoculated with AS5 and AS52 isolates were not associated with the increase in superoxide anion and cell death (Figure 9A–C).

After Ni application, H_2_O_2_ accumulation significantly increased in controls and AS5- and AS52-inoculated plants (Figure 9A). Additionally, Arabidopsis leaves stained with NBT (for superoxide anion detection) and Evans Blue (for cell death exposure) showed intensive staining in Ni-treated plants as compared with non-treated ones and AS8-inoculated individuals (Figure 9B,C).

### 2.10. Effect of Rhizobial Inoculants on the Activity of Antioxidant Enzymes and Phenolic Accumulation under Normal versus Nickel-Stress Conditions

**Figure 10:**
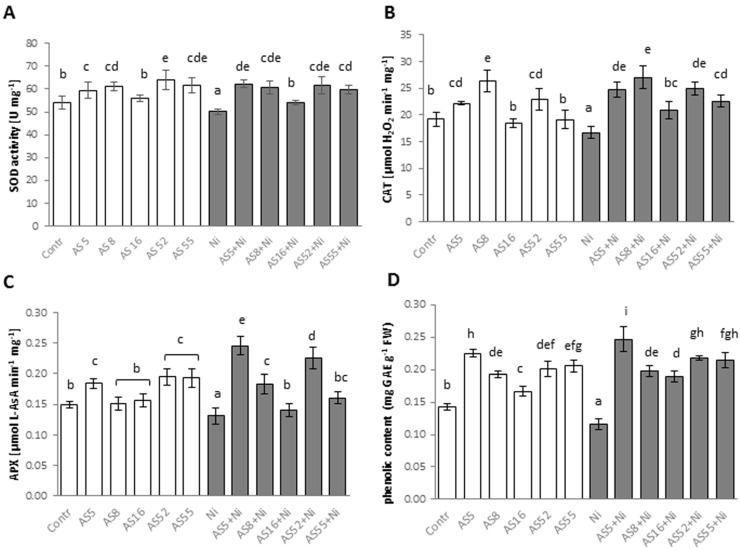
The activities of selected antioxidant enzymes and phenolic compound content in 4-week-old Arabidopsis plants, non-exposed (bars in white) and exposed to 200 µM Ni (bars in grey), in the absence or presence of rhizobial strains. (**A**) SOD, superoxide dismutase activity; (**B**) CAT, catalase activity; (**C**) APX, ascorbate peroxidase activity; (**D**) phenolic content. Values are means (±SD) of 10 plants per isolate from two independent experiments. The same letters indicate the homogenous groups according to the Tukey HSD test at the level *p* = 0.05.

All rhizobial strains (except for AS16) significantly increased SOD activity in Arabidopsis leaves (Figure 10A). The activity of CAT in the leaves of the not-treated Arabidopsis was significantly increased by AS5, AS8, and AS52 strains (Figure 10B), while APX activity significantly increased following AS5, AS52, and AS55 application (Figure 10C). All applied rhizobia enhanced also total phenolic content (Figure 10D).

Nickel treatment decreased SOD activity and phenolic accumulation in non-inoculated controls (Figure 10A,D). In Ni-stressed plants, the use of all rhizobia strains resulted in a significant increase in the activity of SOD, CAT, and APX (Figure 10A–C). The content of total phenolics also significantly increased after all rhizobia applications (Figure 10D).

### 2.11. Arabidopsis Response to Inoculation with Rhizobial Strains and Ni-Stress—Results of the PCA

**Figure 11:**
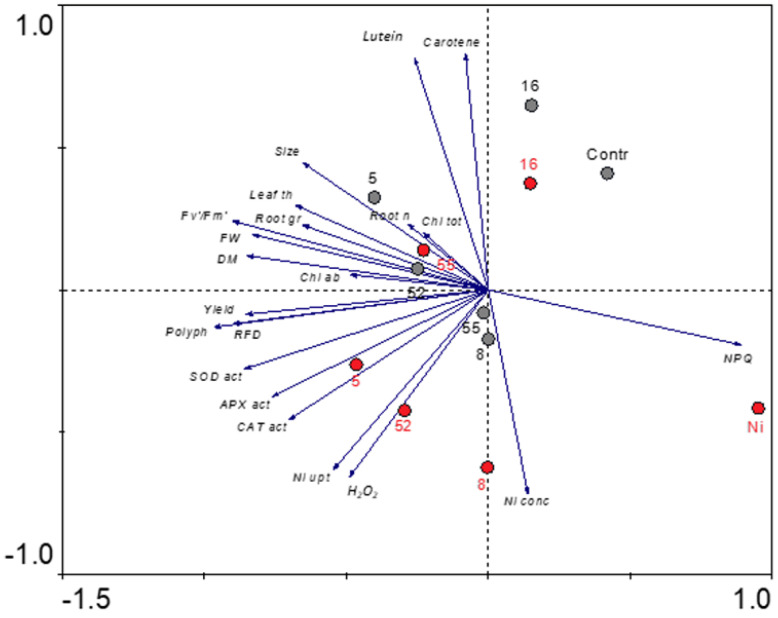
Principal component analysis (PCA) ordination biplot based on Arabidopsis response to inoculation with rhizobia strains and Ni-stress. Vectors indicate the response parameters and circles mark the rhizobia strains: without Ni (grey), with Ni (red). Values next to the axes represent the percentage of explained variability.

The principal component analysis (PCA) showed that the first ordination axis was strongly determined by the following variables: polyphenols, Fv/Fm, Rfd, NPQ, SOD, yield (ϕPSII), dry mass (DM), fresh weight (FW), APX, CAT, leaf thickness, and rosette size (Figure 11). The linear correlation coefficient (r) for these variables ranged from 0.97 (polyphenols) to 0.65 (rosette size), respectively. NPQ correlated negatively, especially with Fv/Fm, yield, DM, and FW. The first axis can be interpreted as a coordinate exhibiting plant growth parameters, photosynthesis efficiency, and antioxidant defense system activity. The second axis mainly represented the gradient of Ni and H_2_O_2_ concentration as well as Ni uptake (r = 0.72, 0.66, and 0.63, respectively) and, on the opposite side, carotene and lutein content (r = 0.83 and 0.81, respectively). The factors exhibiting stress intensity negatively correlated with carotenoid content. The remaining variables including lateral root number, chlorophyll a/b ratio, and total chlorophyll content were insignificant in the obtained model. Based on the position of vectors in the ordination space we could predict a strong, positive correlation between polyphenol content and photosynthesis efficiency, as well as plant growth parameters, especially polyphenols and Rfd, yield, DW, FW, and Fv/Fm. Similarly, a strong positive correlation can be approximated for Ni uptake and H_2_O_2_ content, as well as the activity of antioxidant enzymes, especially catalases.

Projection of sample points to vectors in the ordination space enables one to obtain the fitted abundance values of variables in these samples. Of the plants not exposed to Ni, those inoculated with AS5 isolate showed the most intense growth and photosynthesis efficiency. The AS52 strain also exhibited high PGP abilities, while in AS55 and AS8 strains they were moderate. The plants inoculated with AS16 isolate had the highest lutein and carotene content, whereas the plants exposed to AS8 and AS55 isolates demonstrated very low carotenoid levels. The control plants were characterized by very high NPQ values and very low parameters of plant growth and photosynthesis efficiency.

Under Ni-stress, the plants inoculated with AS5 and then AS52 isolate were distinguished by the highest antioxidant defense system activity, photosynthesis efficiency, and the most intense growth, but they also had high H_2_O_2_ levels (both AS5 and AS52) and accumulated high amounts of Ni (AS52). The AS55 strain demonstrated very high PGP abilities but the inoculated plants had low levels of ROS and antioxidant system activity. The plants inoculated with AS8 isolate had the highest values of stress factors, high antioxidant system activity, and the lowest carotenoid content. The plants growing with AS16 rhizobia strain were characterized by the highest lutein and carotene content and had low levels of stress factors and low antioxidant system activity. Non-inoculated plants exposed to Ni were distinguished by the highest NPQ levels and the lowest values of plant growth and photosynthesis efficiency parameters.

## 3. Discussion

The main factors that limit plant growth on ultramafic soils are Ni contamination and nutrient deficiency. Rhizobia are the most beneficial group of bacteria mainly due to their ability to supply plants and soil with nitrogen during their symbiosis with legumes. This feature is crucial especially on poor, metal-polluted soils [12,13,14]. Moreover, many reports have shown that rhizobia can also interact with non-legume plant species (e.g., *Arabidopsis thaliana*) and promote plant growth [19,46,47,48,49,50,51,52,55]. During this interaction the rhizobia can epiphytically colonize Arabidopsis roots [26,56]. Despite a large number of studies, little is known about the mechanisms of the interactions that result in plant growth promotion, especially under stressful conditions, such as the presence of toxic metals. Due to the natural presence of Arabidopsis on ultramafic dry grasslands [53], we investigated the role of rhizobia nodulating *Anthyllis vulneraria* on ultramafic soil in improving Arabidopsis growth and tolerance to nickel stress.

### 3.1. Plant Growth-Promoting Rhizobia Nodulating Anthyllis Vulneraria on Ultramafic Soil

*A. vulneraria* L. is one of the most important legumes for isolation of rhizobia that promote metal tolerance of the host plant [12,29]. The presented research showed that *A. vulneraria* spontaneously colonizing ultramafic soils formed effective symbiosis with rhizobia that belong to two different taxonomic groups, *Rhizobium* (isolates AS5, AS8, AS16, and AS52) and *Bradyrhizobium* (isolate AS55) according to *glnII* gene sequence analysis (Table 1, Supplementary Figure S1). The isolates showed good growth on the Yeast Extract Congo Red Agar medium and microscopic investigation revealed that they were Gram-negative and rod-shaped (Table 2, Supplementary Figure S3). Moreover, *Rhizobium* sp. (AS5) and *Bradyrhizobium* sp. (AS55) showed high tolerance to nickel (up to 2000 µM) (Table 2, Figure 1). Studies on metal-contaminated sites confirmed that *A. vulneraria* can form symbiotic associations with metal-tolerant *Mesorhizobium* sp., *Bradyrhizobium* sp., and *Rhizobium* sp. [12,57,58]. Our previous studies on metalliferous waste confirmed the presence of effective legume-rhizobia symbiosis with the participation of rhizobia characterized by high tolerance to metals [12,13,14]. The plants growing on ultramafic soils, as well as their associated microsymbionts must have evolved mechanisms to cope with toxic levels of metals present in the soil [13,14]. Moreover, the rhizobia isolated from natural metal-contaminated sites show higher tolerance to metals than the bacteria from non-polluted habitats [29,59,60]. It was shown that Ni-resistance of rhizobia is due to an inducible efflux system, mediated by ATP binding cassette (ABC) transporters, that enables them to lower the intracellular Ni concentration in the cytoplasm [59,61]. We further investigated the Ni-resistant rhizobia for plant growth promotion (PGP) ability and possible stimulation of Arabidopsis growth under Ni stress.

Plant growth-promoting bacteria (PGPB) may directly facilitate plant growth and development. In this study, all tested Anthyllis-nodulating rhizobia showed PGP ability and may directly promote Arabidopsis growth (Table 2). All tested rhizobia produced plant growth hormones (IAA, auxin) and ACC deaminase, and facilitated the acquisition of nutrients (N, Fe, and P) through the production of ammonia, siderophores, and phosphate solubilization mechanism, respectively (Table 2). These rhizobial traits have been suggested as important PGP mechanisms underlying plant growth promotion [13,16,17,18,27,29,30,62]. Root growth and development is regulated by external and internal factors (e.g., phytohormones, auxin). Auxin (IAA) plays a critical role in the regulation of root system architecture by controlling primary root elongation and lateral root formation [63]. In Arabidopsis roots, external IAA application can substantially stimulate primary root elongation [64], while the acropetal transport of IAA in the root is involved in the regulation of root branching [65]. Many rhizobia synthesize IAA [66]. In this study, the tested rhizobia synthesized IAA when supplemented with tryptophan in vitro. Zhao et al. [55] showed that *Rhizobium* sp. IRBG74 can utilize tryptophan secreted from Arabidopsis roots to synthesize IAA. In the case of ethylene, high endogenous ethylene level has a negative effect on the growth and development of plants [67]. Rhizobia with the ability to synthesize ACC deaminase that cleaves ACC (1-aminocyclopropane-1-carboxylate) into α-ketobutyrate and ammonia, reduce the level of ethylene in the plants, which in turn promotes plant growth [68,69]. In this study, all the rhizobial strains produced ACC deaminase, which may stimulate the growth of root system. Moreover, rhizobia may enhance direct Arabidopsis growth by improving the uptake of nutrients (N, P, and Fe). In our study, all the tested free-living rhizobia showed positive results for ammonia production. Other studies confirmed this rhizobial ability [28,31]. Additionally, it was reported that the accumulation of ammonia in soil by PGPB reduces the development of pathogens [70]. Apart from nitrogen, phosphorus is the most limited nutrient for plant growth and the level of soluble phosphate in the ultramafic soil is very low, so bacterial ability to solubilize phosphate is an important mechanism of P acquisition [1,2]. In the present investigation, Anthyllis-nodulating rhizobia from ultramafic soil were capable of solubilizing phosphate, which is in agreement with other studies [13,29,70]. Rhizobia can release the enzymes (e.g., acid phosphatases) that play a major role in mineralization of organic P in the soil and acidify the rhizosphere to stimulate P solubilization [13,16,17,18,19,20,27,29,30,71]. All tested rhizobia produced also siderophores for iron mobilization. Most rhizobia can secrete siderophores to chelate iron [13,16,17,18,19,20,27,29,30,71], but also siderophores can bind metal cations thus limiting their mobility [29,32]. As nutrients become more accessible to roots, they may promote Arabidopsis root and shoot elongation and their biomass. However, growth promotion influenced by PGP rhizobia under in vitro conditions needs to be confirmed in the field.

### 3.2. Arabidopsis Response to PGP Rhizobia Inoculation under Control Conditions

The principal component analysis (PCA) revealed a strong positive correlation between plant weight, rosette size, root growth, leaf thickness, and photosynthesis efficiency parameters. Indeed, the plants inoculated with *Rhizobium* sp. (AS5) and *Bradyrhizobium* sp. (AS55) had the highest values of growth and photosynthesis parameters, whereas the plants inoculated with AS5 and AS16 strains produced significantly bigger rosettes. Moreover, *Rhizobium* sp. (AS5 and AS52) and *Bradyrhizobium* sp. (AS55) significantly increased fresh and dry weight and root growth, while *Rhizobium* sp. (AS8) and *Bradyrhizobium* sp. (AS55) strains were the most effective in root branching. The enhanced development of the above-ground parts stimulates the root development to enhance the range of the penetrated soil area to meet higher requirements of the plant [71]. As a result, plants can access more nutrients from the soil. This is in agreement with other studies on Arabidopsis, where generally rhizobia positively impacted root mass, length, and primary and lateral root development and/or shoot growth [13,24,26]. In the present investigation, IAA producing rhizobia enhanced primary and lateral root development. The production of IAA and gibberellin was described as the main reason for increasing non-legume plant growth and productivity caused by rhizobia application [72]. Additionally, rhizobia (especially AS5 and AS55) inoculation in this study stimulated expression of genes involved in auxin signaling (*IAA12*) and gibberellin biosynthesis (*GA 20-oxidase*) in Arabidopsis. Poitout et al. [26] revealed that *Mesorhizobium loti* enhanced Arabidopsis growth through changes in plant auxin levels. *Rhizobium* sp. IRBG74 promoted the lateral root formation, but inhibited the main root elongation of Arabidopsis by changing auxin signaling [55]. Zhao et al. [55] observed that expression of genes mainly involved in auxin signaling is altered in response to colonization by *Rhizobium* sp. IRBG74. On the other hand, gibberellins regulate germination, stem elongation, and flowering, and synthesis of gibberellins by rhizobia was also documented [73]. Thus, the upregulation of plant hormone genes (auxin and gibberellins) by rhizobia may contribute to better plant growth.

Anatomical studies of leaves are important in assessing the effects of plant growth-promoting organisms. In our study, rhizobia inoculation changed the anatomical organization of Arabidopsis leaves (Table 3). Inoculation with rhizobia, especially *Rhizobium* sp. AS5 and AS16, increased the leaf thickness and the stomata frequency in both epidermis layers. Leaf thickening was associated with the formation of longer mesophyll cells as well as thicker spongy parenchyma, which was especially significant in AS5 inoculated plants (Figure 6, Table 3). The changes in the leaves may be related to higher photosynthetic efficiency. To our knowledge, these rhizobial-induced anatomical changes in Arabidopsis leaves were observed for the first time.

Solar energy absorbed by the chloroplast pigments is used to trigger the photochemical reactions of photosynthesis. Changes in the content of photosynthetic pigments and starch accumulation in chloroplasts are associated with the productivity of photosynthesis [74,75,76,77]. Our study demonstrated that in the rhizobia-treated plants (especially *Rhizobium* sp. AS5 and AS52 strains) the photosynthetic chlorophyll a/b ratio and starch accumulation in chloroplast was higher than in the non-inoculated plants. For *Rhizobium* sp. (AS16) inoculated plants we observed significantly higher carotenoids content than in the non-inoculated control. These results are in accordance with other studies on PGPB application [75,76]. The increase in chlorophyll a/b ratio and pigment content may be due to increased iron and phosphorus uptake due to PGP rhizobia activity, as these nutrients are necessary for the chloroplast formation, the photosynthetic pigment production, and photosynthesis [74,75].

Various chlorophyll *a* fluorescence parameters provide information on the functionality of the photosystem II (PSII) and the efficiency of the photosynthetic apparatus [74]. Chlorophyll fluorescence, similarly as the energy dissipation mechanism in the form of heat, is used to disperse the excess of absorbed light energy and to protect the damage-sensitive components of the photosynthetic apparatus [74]. The Fv/Fm, maximum photochemical efficiency of PSII describes the photochemical activity of the photosynthetic apparatus. The fluorescence parameter Rfd defines plant vitality and photosynthetic capacity of the leaves. Based on the quantum yield of PSII (ϕPSII), the ratio of the quanta used in the photochemical transformations to the total number of absorbed PAR quanta is determined. The NPQ (non-photochemical fluorescence quenching) parameter indicates the effectiveness of photoprotection mechanisms and is associated with the dissipation of excessive excitation energy in the form of harmless heat [74]. In this study, *Rhizobium* sp. strains (especially AS5 and AS52) increased maximum quantum yield of photosystem II (PSII) photochemistry (Fv/Fm), the vitality index (Rfd), and the steady-state quantum yield of PSII photochemistry (ϕPSII) in Arabidopsis leaves. Furthermore, these two strains with the best PGP abilities also slightly lowered the values of non-photochemical quenching (NPQ). The PCA confirmed a negative correlation between the parameters of plant growth and photosynthesis efficiency and the NPQ. The high values of Fv/Fm, RFD, yield, and low NPQ following the rhizobia application indicated high efficiency of photosynthesis. Chlorophyll fluorescence data confirmed the beneficial effect of rhizobia inoculation on the functionality of the photosystem II (PSII) and the photosynthetic apparatus, and these findings corroborated those from other studies [20,23,24,78].

Surprisingly in the present study, we found that rhizobia inoculation (especially with AS5, AS52, and AS55 strains) significantly increased ROS accumulation in Arabidopsis leaves under control conditions. However, this increase was not connected with oxidative stress manifested as cell death. At the same time, we observed high activity of antioxidant enzymes, such as SOD, CAT, and APX and phenolic accumulation. These are all compounds that remove excessive ROS as components of the antioxidant system. The increased activity of SOD that converts superoxide radicals into less toxic H_2_O_2_ resulted in the increase of hydrogen peroxide content in the rhizobia-inoculated plants. On the other hand, CAT dismutates H_2_O_2_ into water and molecular oxygen, while APX converts H_2_O_2_ into water [48]. Additionally, many reports showed that ROS play a dual role in plant physiology [45,46,47,48]. They are important for plant signaling, controlling processes such as growth, development, plant-microbes interactions, but they also induce oxidative damage under stress conditions, when a delicate balance between ROS production and elimination is disturbed [45,46,47,48]. The fact that we did not observe cellular damage and cell death caused by ROS excess in inoculated Arabidopsis plants confirmed the effectiveness of the antioxidant system. On the other hand, it cannot be ruled out that the increase in ROS is the result communication between the rhizobia and the new host. ROS increase in the presence of rhizobia was observed at early steps of their symbiotic interactions with plants [45,46,79].

### 3.3. Arabidopsis Reactions to Rhizobia Inoculation under Ni-Stress

Under nickel excess, the dry mass of *Arabidopsis* plants significantly decreased. Ni stress also inhibited the root growth and lateral root formation. It seems that the growth of Arabidopsis root was more affected than shoot growth under Ni stress. The inhibitory effect of nickel on Arabidopsis growth was confirmed by other studies [39,80]. In this study, inoculation with rhizobia (AS5, AS16, AS52, and AS55) increased the rosette size and fresh and dry weight of Arabidopsis plants, while inoculation with AS5, AS52, and AS55 strains alleviated negative Ni effects on root growth and lateral root formation. Due to positive effects of rhizobia application on Arabidopsis growth under Ni stress, we also tested whether the bacteria induce overexpression of genes associated with biosynthesis of phytohormones that are known plant growth regulators and that could improve Arabidopsis growth and tolerance to Ni stress. We showed that the expression of *IAA12* and *YUC2* genes as well as of *GA 20-oxidase* gene was induced by AS16, AS52, and AS55 strains under Ni stress, while *Rhizobium* sp. (AS5) inoculation induced *GA 30-oxidase* expression in Ni-treated plants. This suggested possible role of these genes in Arabidopsis tolerance to Ni stress. Our results for rhizobia corroborated those obtained in other studies. Upregulation of AUX/IAA1 (transcriptional repressor of auxin responsive gene) genes following inoculation with PGPB under stress conditions was also been demonstrated [81]. Under metal stress, the growth promotion stimulated by rhizobia is mainly due to counteracting biomass decrease thanks to improved mineral nutrition, secretion of phytohormones, enhancing photosynthesis efficiency, and stimulating antioxidant activity [13,16,17,18,19,20,28,30].

In our study, Ni exposure lowered Fv/Fm, Rfd, and ϕPSII parameters, while enhanced NPQ value in Arabidopsis leaves, thus indicating stress-induced photoinhibition and PSII damage [36,39,74,82,83]. Stress-induced photoinhibition in plants results in a decrease in Fv/Fm [74,82,83]. The response of the plant photosynthetic apparatus to high concentration of heavy metals in the substrate is most often manifested by a decrease in photochemical quenching and lower electron transport across a PSII. Simultaneous increase in NPQ serves as a protection mechanism against excessive light energy that which the plant cannot use, because the excess of heavy metals limits the performance of the photosynthetic apparatus, e.g., by reducing the amount of chlorophyll [74,82,83]. In this study, Rfd decrease in comparison with non-treated plants indicated disturbances in the course of photochemical reactions in the thylakoids and slow rate of enzymatic reactions in the chloroplast stroma [74,82,83]. Under metal stress, the decrease in the yield (ϕPSII) parameter is caused by closing of the stomata, which reduces the amount of assimilated CO_2_, and possibly by a decrease in the fluidity of thylakoid membranes [74]. Under Ni stress, reduced efficiency of photosynthesis and the increase in the NPQ value indicated the PSII damage. These stress-induced changes in the parameters of chlorophyll fluorescence were associated with reduced leaf thickness and pigment content, as well as deformations in the chloroplast structure. Additionally, Ni application reduced chlorophyll a/b ratio and pigment content in Arabidopsis leaves. Our results were similar to those from other studies on heavy metal toxicity [36,39,74,82]. For example, Ni was shown to damage the photosynthetic apparatus, destruct mesophyll cells and the structure of thylakoid membranes, and decreases chlorophyll content [36,82]. The drop in photosynthetic pigments could be a consequence of nutrient (P, Fe, and Mg) deficiency caused by the inhibition of root growth and/or an elevated ROS production at Ni excess [84]. We showed that the application of AS5 and AS52 rhizobia alleviated the detrimental effects of Ni stress on photosynthesis. Inoculation with these strains under Ni stress increased Fv/Fm, Rfd and ϕPSII values, while decreased the NPQ. Other studies showed that under metal stress PGPB inoculation improved the photochemical apparatus integrity and functionality [85]. Moreover, the alleviation of the damaging effects of Ni on photosynthesis by *Rhizobium* sp. (especially AS5 and AS52) was associated with higher total chlorophyll content and chlorophyll a/b ratio in inoculated plants, and in the case of *Rhizobium* sp. (AS16) with higher carotenoid content. Moreover, the light microscope and TEM observations confirmed that Ni stress decreased leaf thickness and destroyed the chloroplast ultrastructure by degradation of internal chloroplast membranes and lowering the amount of accumulated starch. These chloroplast deformations where often observed in Ni-treated plants [82]. In our study, rhizobia reduced Ni toxic effect in Arabidopsis leaves. Rhizobia induced leaf thickening and formation of longer palisade cells, as well as wider spongy mesophyll with enlarged air chambers to facilitate gas exchange, which was especially significant in AS5 and AS52 inoculated plants. The observed leaf properties may be related to the higher number of chloroplasts and hence greater photosynthetic pigment content, as well as better delivery of carbon dioxide for photosynthesis. Thus, these results signified that PGP rhizobia counteracted the negative effects of Ni stress on photosynthetic activity, resulting in a positive effect on plant growth.

In this study, Ni induced ROS accumulation and cell death in Arabidopsis leaves. Simultaneously, Ni-treated plants showed low phenolic content and decreased activity of SOD. Hydrogen peroxide is a signaling molecule at low concentration, but at higher concentration it causes oxidative burst in the cells and initiates protein oxidation, promotes lipid peroxidation, DNA damage, and plant cell death [46,47,48]. Overproduction of ROS is one of the first cellular response to toxic metals. Toxic metals can activate ROS production in the apoplast and in the organelles, such as the chloroplasts and mitochondria [86]. Cellular ROS homeostasis is essential for plants due to the dual role of ROS. Plants are equipped with a complex antioxidant system regulating the balance of the cellular redox potential especially under stress conditions. In this study, the PCA showed that Ni-treated plants inoculated with *Rhizobium* sp. (AS5 and AS52) exhibited high antioxidant defense system activity, but they also had high content of H_2_O_2_ (for both strains) and Ni concentration and uptake (strain AS52). The studied rhizobia enhanced the activity of SOD, CAT, and APX and phenolics accumulation in Ni-stressed plants, and these compounds could scavenge the excessive ROS. Our results corroborated those of other studies. Carotenoids and phenols play important roles in protecting thylakoid membranes and other cellular membranes from lipid peroxidation [46]. Ju et al. [87] showed a similar positive effect of rhizobia (*Sinorhizobium meliloti*) in Cu-stressed *Medicago sativa*, where a remarkable decline in the production of ROS and increased activity of SOD, CAT, and APX were observed. It seems that inoculation of Arabidopsis with Ni-tolerant rhizobia activated the plant antioxidant system, which is essential to limit the oxidative stress induced by the metal.

In the present study, we observed a weak promoting effect of *Rhizobium* sp. (AS8 strain) on Arabidopsis growth. However, Ni-tolerant AS8 stimulated the lateral root formation and Ni uptake in Arabidopsis. It seems that plants could not cope with high toxicity of Ni, which was reflected in their poor growth, low starch and carotenoid content, and decrease in photosynthesis efficiency. The plants inoculated with *Rhizobium* sp. (AS8) showed high activity of antioxidant enzymes (especially CAT), but it was insufficient to counteract the oxidative stress induced by Ni. Aqeel et al. [88] confirmed that plant growth inhibition may be the consequence of Ni uptake by plants, which prevented them from obtaining nutrients required for physiological functions and reduced photosynthesis.

## 4. Materials and Methods

### 4.1. Study Site and Samples Collection

*Anthyllis vulneraria* L. plants were collected in June 2018 from ultramafic tailings located on the site of a former nickel ore mine near the village of Szklary, Lower Silesia region, SW Poland (50°38′81.4″–50°38′83.2″ N, 16°50′21.2″–16°50′20.9″ E). A detailed description of the study area and ultramafic rocks characteristic were presented in our earlier work [13], where we showed that Ni was the significant and predominant metal pollutant in this ultramafic soil (Ni content 1500 mg kg^−1^).

Anthyllis plants together with the rhizosphere soil (at a depth of 0–30 cm) were collected in the flowering phase from three sample sites, where they dominated the vegetation cover. The samples were wetted with tap water, packed in plastic bags and transported to the laboratory as soon as possible to maintain the viability of the bacteria. In August 2018 we also collected seeds from the same sites for authentication tests.

### 4.2. Rhizobia Isolation, Identification and Characterization

For rhizobia isolation, about twenty pink (effective) *A. vulneraria* nodules were randomly selected and grown on Yeast Extract-Mannitol (YEM) medium according to Vincent [89] method. For rhizobia molecular identification, the Prep-Man^®^ Ultra Sample Preparation Reagent (Applied Biosystems, Foster City, CA, USA) was used for the DNA extraction following the procedure recommended by the manufacturer and seventy strains were initially characterized according to BOX1AR (5′ CTACGGCAAGGCGA CGCTGACG 3′) patterns, which were obtained following the protocol of Versalovic et al. [90]. We selected six isolates (AS5, AS8, AS14, AS16, AS52 and AS55) representing different BOX-PCR profiles for identification according to *glnII* gene amplification. Total genomic DNA was isolated from fresh pure cultures. The amplification of partial sequences (519 bp) of *glnII* gene (encoding glutamine synthetase) was carried out following the protocol reported by Banasiewicz et al. [54], using TSglnIIf (5′ AAG CTC GAG TAC ATC TGG CTC GAY GG 3′) and TSglnIIr primers (5′ SGA GCC GTT CCA GTC GGT RTC G 3′). The DreamTaqTM DNA Polymerase (Thermo Fisher Scientific, Waltham, MA, USA) has been applied in all amplification assays. Amplicon sizes were estimated using 1% agarose gel electrophoresis, purified with the with the E.Z.N.A. Gel Extraction Kit (Omega Bio-tek^®^, Norcross, GA, USA) and sequenced on an ABI3100 Automated Capillary DNA sequencer (Applied Biosystems, Foster City, CA, USA). Sequence chromatograms were visualized and manually edited using BioEdit 7.2.3, MultAlin and subjected to BLASTn searches to compare them to the nucleotide sequences stored in GenBank [https://www.ncbi.nlm.nih.gov (accessed on 24 June 2022)]. The *glnII* sequences were aligned using ClustalW software and maximum likelihood (ML) phylogeny was inferred with Mega 6 using the best-fit nucleotide substitution model, GTR + I + G, as indicated by jModelTest 2.1.4. Branch support was estimated using non-parametric bootstrap analyses based on 500 replicates. The ML *glnII* tree is shown in Supplementary Figure S2. Nucleotide sequence data reported are available in the NCBI GenBank database under the accession numbers ON838933–ON838937.

The nodulation ability of AS5, AS8, AS16, AS52 and AS55 strains were performed in the authentication test on Anthyllis plants on sterile ultramafic soil according to our previous work [12]. Additionally, these strains were performed morphological and biochemical characterization on selective for rhizobia Yeast Extract Congo Red Agar medium, and gram staining according to Debojyoti et al. [91]. Further these rhizobial strains were characterized for their plant growth promoting (PGP) traits using specific media. Ammonia production was assayed with Nessler reagent and development of slight yellow to brownish color was considered to be a positive test for ammonia production [91], which production was categorized on the basis of color intensity. Phosphate solubilization was assayed on plates with Pikovskaya’s agar medium and the formation of zone of clearance around the colonies indicated the positive solubilization ability and solubilization potency was measured based on Phosphate Solubilization Index (PSI) as Total diameter of halo zone/Colony diameter [92,93]. Siderophore release was determined with Chrome Azurol S (CAS) agar medium. Synthesis of indole acetic acid (IAA) was performed on Luria-Bertani broth (LB) and Salkowski reagent according to Gordon and Weber [94] and expressed as μg IAA mL^−1^. The determination of 1-aminocyclopropane-1-carboxylate (ACC) deaminase activity in the strains was evaluated according to Penrose and Glick [95] and unit of ACC deaminase activity was expressed as the amount of α-ketobutyrate produced in µmol per microgram of cellular protein/hour. All sample measurements were performed in triplicate.

### 4.3. Rhizobia Tolerance to Nickel

The ability of Anthyllis-nodulate rhizobial strains to grow in the presence of nickel were tested on SLP agar plates (not shown) and liquid SLP medium according to our earlier studies [13]. 1 mM stock solution of NiSO_4_ sterilized by filtration (0.20 μm) was added to sterile SLP medium as 200 µM or 2000 µM Ni. These metal doses were selected based on our previous study and the reactive forms in the examined ultramafic soil [13]. The strains were grown in liquid SLP medium supplemented with Ni 200 µM or 2000 µM for 48 h at 28 °C and 100 μL of 10^8^ cell/mL bacterial culture were deposited onto SLP agar plates and incubated at 28 °C for 24–72 h. The analysis in liquid SLP medium was carried out with the same Ni concentrations and the bacterial growth was estimated by measuring optical density (OD) at 600 nm using a Biospectrometer (Eppendorf). Three replicates were made for each bacterial strain and each test was repeated two times.

### 4.4. Arabidopsis Root Growth Promotion under Ni Stress on Vertical Agar Plates

The AS5, AS8, AS16, AS52 and AS55 strains were inoculated onto agar plates with *Arabidopsis thaliana* Columbia-0 (Col-0) seedlings to study root growth responses in the presence of Ni. The seeds were surface sterilized for 7 min with a 1:1 solution of domestos and water and washed 20 min with distilled water. Subsequently, the seeds were put on vertical placed agar plates with Gamborg medium and left to grow for 5 days in following conditions (long photoperiod 16/8 day/night, 80–100 µE m^−2^s^−^^1^ lighting, 25 °C and humidity 50%. Next the seedlings were transferred to fresh Gamborg vertical placed plates (5 plants per plate) supplemented with Ni (0; Ni 200 µM as NiSO_4_) and were or not inoculated with 10 μL of freshly prepared bacteria cultures (optical density of 0.2 at 600 nm). The Ni concentration was selected based on our previous study and the total and/or the reactive forms in the examined ultramafic soil [13]. The experiment was laid out in three replicates for each treatment. We measured length of primary root and number of lateral roots formation after 5 days of growth.

### 4.5. Jiffy Pots Experiment

The seeds of Arabidopsis were sown individually in Jiffy pots and half of them were inoculated with rhizobial strains (AS5, AS8, AS16, AS52, AS55; 0.5 mL of liquid YEM culture per pot, with optical density of 0.2 at 600 nm). Un-inoculated control Arabidopsis plants were treated in a similar way with sterile YEM medium. For each strain 20 pots were prepared. Plants were grown for 4 weeks in laboratory conditions (8 h photoperiod, 80 µE m^−2^s^−1^ lighting, 50% relative air humidity, and temperature day/night: 22/18 °C). Plants were watered every two days with tap water or 200 µM Ni (1 mL per pot). After this time, the rosettes were cut and weighed. The rosette size was analyzed with a FluorCam (Photon System Instruments PSI, Brno, Czech Republic) for 10 plants per isolate in two independent experiments. The dry weight of whole rosettes was measured after three-day-long desiccation in 105 °C from 12 plants per treatment from at least two independent experiments.

### 4.6. Analysis of Ni Concentration

Shoots of 4-week old Arabidopsis were dried at 40 °C and ground. Than the plant material was oven ashed at 475 °C overnight. The ash was dissolved in 6M HCl and Ni concentration was determined with a Varian SpectrAA 220FS AAS apparatus [13]. All analysis and measurements were performed in triplicate.

### 4.7. Examination of Leaf Anatomy and Chloroplast Ultrastructure

Fully expanded leaves of 4-week old Arabidopsis plants were processed for anatomical and ultrastructural assays. Small fragments of leaves were fixed, embedded in Epoxy resin and cut into semi-thin and ultra-thin sections according to our previous study [13]. The semithin sections were stained with 1% toluidine blue and observed under light microscopy (Olympus-Provis, Tokyo, Japan) and ultrathin sections collected on formvar-coated grids were short stained with uranyl acetate and lead citrate and examined under a transmission electron microscope (Morgagni). All images were saved as .jpg files and if necessary were adjusted using Photoshop CS 8.0 (Adobe Systems, San Jose, CA, USA) software by non-destructive tools (contrast and/or levels).

### 4.8. Photosynthetic Pigments Composition and Chlorophyll a Fluorescence

The photosynthetic pigments content were determined as precisely described [96]. Pigments extracted from 4-week old Arabidopsis plants (previously grinded in liquid nitrogen and stored in −80 °C) were eluted on Synergi™ 4 µm Max-RP 80 Å, LC Column 250 × 4.6 mm (Phenomenex, Torrance, CA, USA), at 30 °C for 20 min and flow rate 1 mL min^−1^, using Shimadzu HPLC System (Shimadzu, Kyoto, Japan). Results were expressed as peak area per µg of fresh weight.

The Closed FluorCam FC 800-C System (PSI, Drásov, Czech Republic) was used to measure of chlorophyll a fluorescence and rosette size. Quenching protocol was performed on 30 min dark-adapted plants due to determine Fv/Fm, Rfd, ϕPSII and NPQ parameters [83]. Measurements were performed for 12 plants per each treatment.

### 4.9. Hydrogen Peroxide Accumulation and ROS, and Cell Death Localization

Hydrogen peroxide (H_2_O_2_) accumulation in cells was spectrophotometrically determined according to Rusaczonek et al. [96] for 12 plants per each treatment. For superoxide detection Arabidopsis leaves where stained with Nitro blue tetrazolium (NBT) according to Rusaczonek et al. [96]. Superoxide production was visualized as a dark blue formazan deposit within the leaf tissue. For the visualization of cell death, leaves were stained with 1% (*m*/*v*) Evans blue and vacuum infiltrated for 30 min and leaved for 8 h incubation at room temperature [97]. Next, all leaves were decolorated for two days in 0.25% (*m*/*v*) chloral hydrate. The staining was observed under binocular (Leica M165 FC) and the images were saved as .jpg files.

### 4.10. Enzyme Activity Measurements

For protein extraction, frozen leaf tissue (50–100 mg) was homogenized with extraction buffer using Tissue Lyser MM400 (Retsch, Haan, Germany) as precisely described previously [96] Bradford assay kit (Thermo Scientific, Carlsbad, CA, USA) was used to determine protein concentration. The activity of selected enzymes, such as superoxide dismutase (SOD), catalase (CAT), and ascorbate peroxidase (APX) was spectrophotometrically determined based on the methodology described in earlier work [96]. All measurements were performed for 12 plants per each treatment.

### 4.11. Phenols Concentration

Estimation of total phenolic content was determined using method published by Ainsworth and Gillespie [98] and was expressed as GAE (Gallic Acid Equivalent), i.e., mg of GAE per gram of fresh weight; for 12 plants per each treatment.

### 4.12. RNA Isolation, cDNA Synthesis and qPCR Analysis

Rosettes of Arabidopsis plants were frozen in liquid nitrogen, three individual biological replicates (each containing 10 plants) were collected and stored in −80 °C. Extraction of total RNA was performed using a GeneMATRIX Universal RNA Purification Kit (EURX, Gdańsk, Poland) with an additional step of on-column DNaseI digestion. Concentration and purity of RNA were checked using Eppendorf BioSpectrometer (Eppendorf, Hamburg, Germany), moreover quality of extracted RNA was controlled by electrophoretic separation in 1% agarose gel. cDNA synthesis was performed for equimolar RNA amounts of each sample using a High Capacity cDNA Reverse Transcription Kit (Thermo Fisher Scientific, Carlsbad, CA, USA) according to the manufacture instruction. Real-time PCR experiments were conducted in 96-well reaction plates using a Bio-Rad CFX96 Touch™ Real-Time PCR Detection System (Bio-Rad, Hercules, CA, USA) using iTaq Universal SYBR Green Supermix (Bio-Rad, Hercules, CA, USA). Two genes were used as a reference; 5-FORMYLTETRAHYDROFOLATE CYCLOLIGASE (5-FCL, AT5G13050), and PROTEIN PHOSPHATASE 2A SUBUNIT A2 (PP2AA2, AT3G25800). All primers used in this work are listed in Supplementary Table S1 and were designed in primer 3 software.

### 4.13. Data Analysis

Differences among means in the experimental data were analyzed by the one-way ANOVA. In the case of the rhizobial strains tolerance to different doses of nickel the two-way ANOVA was used. To obtain the homogeneous groups the post-hoc Tukey HSD test was performed. All these analyses were carried out at the level *p* = 0.05 with TIBCO Software Inc. (2017) and Statistica (data analysis software system) version 13.3 (http://statistica.io (accessed on 24 June 2022), permanent license, StatSoft, Poland). To analyse the variance in Arabidopsis reaction to rhizobial strains inoculation and nickel stress, the principal component analysis (PCA) was performed on a correlation matrix. The PCA as a method of ordination were chosen after preliminary application of the detrended correspondence analysis (DCA), which yielded axes of short gradients, i.e., <0.2 SD [99]. Computations and ordination plot of PCA were made using CANOCO 4.55 for Windows software (Wageningen, The Netherlands) [100].

## 5. Conclusions

Our results indicated that the Anthyllis-nodulating selected rhizobia from ultramafic soil showed PGP ability and may directly promote Arabidopsis growth. Under Ni-stress inoculation with *Rhizobium* sp. (AS5 and AS52) and *Bradyrhizobium* sp. (AS55) alleviated the negative effect of Ni and increase Arabidopsis tolerance to Ni. Therefore, native rhizobia isolated from heavy metal-contaminated ultramafic soils have the potential to be applied as PGP bacteria for non-legume plants to allow them to grow on metal-polluted soils and to provide high efficiency for phytoremediation of Ni-contaminated sites.

## Figures and Tables

**Table 1:** Identification based on *glnII* gene sequences of the rhizobia isolates.

Strain ID	Name of Bacteria	Best Match Sequence and Accession Number	Similarity (%)
AS5	*Rhizobium* sp.	*Rhizobium tibeticum* HAMBI 3177 (KF206791.1)	95.38%
		*Rhizobium tibeticum* CCBAU 85039^T^ (EU407190.1)	95.38%
AS8	*Rhizobium* sp.	*Rhizobium tibeticum* HAMBI 3177 (KF206791.1)	95.38%
		*Rhizobium tibeticum* CCBAU 85039^T^ (EU407190.1)	95.38%
AS16	*Rhizobium* sp.	*Rhizobium* sp. RMCC TR2021 (KM881220.1)	100%
AS52	*Rhizobium* sp.	*Rhizobium* sp. GG5/GG20/GG37 (MN026364.1/MN026365.1/MN026366.1)	98.76%
AS55	*Bradyrhizobium* sp.	*Bradyrhizobium* sp. 7Cha14Z (MT790547.1)	99.04%

**Table 2:** Characteristics of rhizobial strains.

Isolate Name/Features	AS 5	AS 8	AS 16	AS 52	AS 55
Growth on Yeast Extract Congo Red Agar medium	positive	positive	positive	positive	positive
Gram staining	pink	pink	pink	pink	pink
Shape	rod	rod	rod	rod	rod
Color	white	white	white	white	white, gummy
Ammonia production *	++	++	+	++	++
Siderophore production	positive	positive	positive	positive	positive
Phosphate solubilization (PSI)	2 ± 0.1 a	2 ± 0.1 a	2 ± 0.1 a	2 ± 0.2 a	2 ± 0.2 a
IAA synthesis (μg/mL + 0.2% tryptophan)	1.8 ± 0.1 a	1.9 ±0.2 a	1.1 ± 0.2 b	2.0 ± 0.1 a	2.1 ±0.2 a
ACC deaminase activity (µmol α-ketobutyrate µg^−1^ protein h^−1^)	0.93 ± 0.01 a	0.90 ± 0.01 a	0.32 ± 0.01 b	0.91 ± 0.01 a	0.92 ± 0.02 a

* The levels of production [low (+) and high (++), according to color intensity] were established. PSI—Phosphate Solubilization Index. The values are presented as means ± standard deviation. Different letters indicate a statistically significant difference among treatments in ANOVA comparisons and the post-hoc Tukey HSD at *p* = 0.05.

**Table 3:** Anatomical traits of leaves of Arabidopsis plants.

Traits/ Bacterial Isolates	Total Thickness (µm)	Number of Palisade Cell Layers	Length of Palisade Parenchyma Cells (µm)	Width of Palisade Parenchyma Cells (µm)	Number of Spongy Cell Layers	Length of Spongy Parenchyma Cells (µm)	Width of Spongy Parenchyma Cells (µm)	Stomata Number in Adaxial Epidermis	Stomata Number in Abaxial Epidermis
Control	122.2 ± 5.3 b	1	35.8 ± 5.6 ab	26.9 ± 1.8 ab	3	24.0 ± 5.4 a	30.2 ± 3.5 a	1.0 ± 0.0 a	1.7 ± 0.5 a
AS5	214.0 ± 3.8 f	1	60.1 ± 4.7 e	51.4 ± 7.5 e	3–5	41.1 ± 6.1 b	53.4 ± 5.7 d	1.6 ± 0.5 ab	2.7 ± 0.8 ab
AS8	141.2 ± 5.79 bcd	1	40.3 ± 5.8 abc	41.4 ± 1.8 d	3–4	25.9 ± 1.8 a	33.1 ± 3.4 ab	1.5 ± 0.5 ab	4.0 ± 0.7 abc
AS16	162.6 ± 14.7 e	1	46.2 ± 3.5 c	40.2 ± 5.8 cd	3–4	27.8 ± 2.0 a	40.0 ± 6.8 abc	2.5 ± 0.8 b	3.3 ± 0.3 abc
AS52	140.5 ± 3.2 bcd	1	44.2 ± 5.3 bc	30.7 ± 2.1 ab	3–4	27.3 ± 4.4 a	34.7 ± 5.7 abc	1.5 ± 0.5 ab	5.0 ± 0.4 bcd
AS55	130.3 ± 5.1 bc	1	36.7 ± 5.0 ab	33.5 ± 2.7 bcd	3–4	25.4 ± 1.7 a	41.8 ± 5.6 a	1.6 ± 0.5 ab	5.6 ± 0.4 bcd
+Ni	115.6 ± 7.4 a	1	32.8 ± 1.2 a	28.7 ± 6.4 ab	3	23.0 ± 3.9 a	44.2 ± 7.1 cd	1.5 ± 0.5 ab	4.0 ± 0.5 cde
AS5 + Ni	160.6 ± 21.9 ef	1	42.4 ± 5.7 bc	23.6 ± 2.8 a	4–5	25.0 ± 2.8 a	36.9 ± 4.5 abc	2.3 ± 1.0 b	4.8 ± 0.5 cde
AS8 + Ni	146.8 ± 7.6 cde	1	45.3 ± 2.5 bc	42.1 ± 6.0 de	3–4	26.6 ± 3.2 a	36.7 ± 3.1 abc	1.8 ± 0.4 ab	4.5 ± 0.3 cde
AS16 + Ni	148.5 ± 17.6 cde	1	45.1 ± 11.6 bc	31.0 ± 5.7 ab	4	27.8 ± 2 a	36.9 ± 6.4 abc	1.8 ± 0.4 ab	3.3 ± 0.2 cde
AS52 + Ni	147.0 ± 3.4 cde	1	36.8 ± 2.7 bc	34.4 ± 4.4 bcd	3–4	28.7 ± 3.9 ab	34.0 ± 3.1 ab	2.2 ± 0.9 ab	2.1 ± 0.9 cde
AS55 + Ni	142.8 ± 5.7 bcd	1	43.7 ± 1.7 bc	34.2 ± 5.4 bcd	3–4	26.0 ± 4.3 a	41.8 ± 5.6 bc	1.5 ± 0.5 ab	5.6 ± 0.3 e

Values are means ± SD of 10 plants per treatment from two independent experiments. Different letters indicate significantly different means at *p* = 0.05 according to one-way ANOVA and post-hoc Tukey’s test.

## Data Availability

The data that support the findings of this study are available on request from the corresponding author. The data are not publicly available due to privacy or ethical restrictions.

## Supplementary Materials

The following supporting information can be downloaded at: https://www.mdpi.com/article/10.3390/ijms231911538/s1.

## References

[1] Brady K.U., Kruckeberg A.R., Bradshaw H.D. (2005). Evolutionary ecology of plant adaptation to serpentine soils. Ann. Rev. Ecol. Evol. Syst..

[2] Kazakou E., Dimitrakopoulos P.G., Baker A.J.M., Reeves R.D., Troumbis A.Y. (2008). Hypotheses, mechanisms and tradeoffs of tolerance and adaptation to serpentine soils: From species to ecosystem level. Biol. Rev..

[3] Brooks R.R. (1987). Serpentine and Its Vegetation: A Multidisciplinary Approach.

[4] Kruckeberg A.R. (2002). Geology and Plant Life: The Effects of Land Forms and Rock Types on Plants.

[5] Proctor J.K. (2003). Vegetation and soil and plant chemistry on ultramafic rocks in the tropical Far East. Perspect. Plant Ecol. Evol. Syst..

[6] Alexander E.B., Coleman R.G., Keeler-Wolf T., Harrison S.P. (2007). Serpentine Geoecology of Western North America: Geology, Soils and Vegetation.

[7] Reeves R.D., Baker A.J.M., Becquer T., Echevarria G., Miranda Z.J.G. (2007). The flora and biogeochemistry of the ultramafic soils of Goiás state, Brazil. Plant Soil.

[8] Rajkumar M., Prasad M.N., Freitas H., Ae N. (2009). Biotechnological applications of serpentine soil bacteria for phytoremediation of trace metals. Crit. Rev. Biotechnol..

[9] Majorel C., Hannibal L., Ducousso M., Lebrun M., Jourand P. (2014). Evidence of nickel (Ni) efflux in Ni-tolerant ectomycorhizal *Pisolithus albus* isolated from ultramafic soil. Environ. Microbiol. Rep..

[10] Vincent B., Juillot F., Fritsch E., Klonowska A., Gerbert N., Acherar S., Grangeteau C., Hannibal L., Galiana A., Ducousso M. (2019). A leguminous species exploiting alpha- and beta rhizobia for adaptation to ultramafic and volcano-sedimentary soils: An endemic *Acacia spirorbis* model from New Caledonia. FEMS Microbiol. Ecol..

[11] Kasowska D., Koszelnik-Leszek A. (2014). Ecological features of spontaneous vascular flora of serpentine post-mining sites in Lower Silesia. Arch. Environ. Prot..

[12] Sujkowska-Rybkowska M., Ważny R. (2018). Metal resistant rhizobia and ultrastructure of *Anthyllis vulneraria* nodules from zinc and lead contaminated tailing in Poland. Int. J. Phytorem..

[13] Sujkowska-Rybkowska M., Kasowska D., Gediga K., Banasiewicz J., Stępkowski T. (2020). *Lotus corniculatus*-rhizobia symbiosis under Ni, Co and Cr stress on ultramafic soil. Plant Soil..

[14] Oleńska E., Małek W., Sujkowska-Rybkowska M., Szopa S., Włostowski T., Aleksandrowicz O., Święcicka I., Wójcik M., Thijs S., Vangronsveld J. (2022). An alliance of *Trifolium repens-Rhizobium leguminosarum* bv. *trifolii* mycorrhizal fungi from an old Zn-Pb-Cd rich waste heap as a promising tripartite system for phytostabilization of metal polluted soils. Front. Microbiol..

[15] Berg G. (2009). Plant-microbe interactions promoting plant growth and health: Perspectives for controlled use of microorganisms in agriculture. Appl. Microbiol. Biotechnol..

[16] Oldroyd G.E., Murray J.D., Poole P.S., Downie J.A. (2011). The rules of engagement in the legume-rhizobial symbiosis. Annu. Rev. Genet..

[17] Shokri D., Emtiazi G. (2010). Indole-3-acetic acid (IAA) production in symbiotic and non-symbiotic nitrogen-fixing bacteria and its optimization by Taguchi design. Curr. Microbiol..

[18] Bal H.B., Das S., Dangar T.K., Adhya T.K. (2013). ACC deaminase and IAA producing growth promoting bacteria from the rhizosphere soil of tropical rice plants. J. Basic Microbiol..

[19] Flores-Felix J.D., Menendez E., Rivera L.P., Marcos-Garcia M., Martinez-Hidalgo P., Mateos P.F., Martinez-Molina E., Velazquez M.D., Garcia-Fraile P., Rivas R. (2013). Use of *Rhizobium leguminosarum* as a potential biofertilizer for *Lactuca sativa* and *Daucus carota* crops. J. Plant Nutr. Soil Sci..

[20] Antoun H., Beauchamp C.J., Goussard N., Chabot R., Lalande R. (1998). Potential of Rhizobium and Bradyrhizobium species as plant growth promoting rhizobacteria on non-legumes: Effect on radishes (*Raphanus sativus* L.). Plant Soil.

[21] Biswas J.C., Ladha J.K., Dazzo F.B. (2000). Rhizobial inoculation influences seedling vigor and yield of rice. Agron. J..

[22] Gutiérrez-Zamora M.L., Martínez-Romero E. (2001). Natural endophytic association between *Rhizobium etli* and maize (*Zea mays* L.). J. Biotechnol..

[23] Franche C., Lindström K., Elmerich C. (2009). Nitrogen-fixing bacteria associated with leguminous and non-leguminous plants. Plant Soil.

[24] García-Fraile P., Carro L., Robledo M., Ramírez-Bahena M.H., Flores-Félix J.D., Fernández M.T., Mateos P.F., Rivas R., Igual J.M., Martínez-Molina E. (2012). Rhizobium promotes non-legumes growth and quality in several production steps: Towards a biofertilization of edible raw vegetables healthy for humans. PLoS ONE.

[25] Yanni Y.G., Dazzo F.B., Squartini A., Zanardo M., Zidan M.I., Elsadany A.E.Y. (2016). Assessment of the natural endophytic association between *Rhizobium* and wheat and its ability to increase wheat production in the Nile delta. Plant Soil.

[26] Poitout A., Martinière A., Kucharczyk B., Queruel N., Silva-Andia J., Mashkoor S., Gamet L., Varoquaux F., Paris N., Sentenac H. (2017). Local signalling pathways regulate the Arabidopsis root developmental response to *Mesorhizobium loti* inoculation. J. Exp. Bot..

[27] Dakora F., Matiru V., Kanu A. (2015). Rhizosphere ecology of lumichrome and riboflavin, two bacterial signal molecules eliciting developmental changes in plants. Front. Plant Sci..

[28] Gopalakrishnan S., Sathya A., Vijayabharathi R., Varshney R.K., Gowda C.L.L., Krishnamurthy L. (2015). Plant growth promoting rhizobia: Challenges and opportunities. 3 Biotech.

[29] Fagorzi C., Checcucci A., di Cenzo G.C., Debiec-Andrzejewska K., Dziewit Ł., Pini F., Mengoni A. (2018). Harnessing Rhizobia to improve heavy-metal phytoremediation by legumes. Genes.

[30] Jaiswal S.K., Mohammed M., Ibny F.Y.I., Dakora F.D. (2021). Rhizobia as a source of plant growth-promoting molecules: Potential applications and possible operational mechanisms. Front. Sustain. Food Syst..

[31] Mir M.I., Kumar B.K., Gopalakrishnan S., Vadlamudi S., Hameeda B. (2021). Characterization of rhizobia isolated from leguminous plants and their impact on the growth of ICCV 2 variety of chickpea (*Cicer arietinum* L.). Heliyon.

[32] Jiang C.Y., Sheng X.F., Qian M., Wang Q.Y. (2008). Isolation and characterization of a heavy metal-resistant *Burkholderia* sp. from heavy metal-contaminated paddy field soil and its potential in promoting plant growth and heavy metal accumulation in metal-polluted soil. Chemosphere.

[33] Costa F.S., Macedo M.W.F.S., Araújo A.C.M., Rodrigues C.A., Kuramae E.E., de Barros Alcanfor S.K., Pessoa-Filho M., Barreto C.C. (2019). Assessing nickel tolerance of bacteria isolated from serpentine soils. Braz. J. Microbiol..

[34] Jing Y., He Z., Yang X. (2007). Role of soil rhizobacteria in phytoremediation of heavy metal contaminated soils. Zhejiang Univ. Sci. B.

[35] Brown P.H., Welch R.M., Cary E.E. (1987). Nickel: A micronutrient essential for higher plants. Plant Physiol..

[36] Ahmad M.S.A., Ashraf M. (2011). Essential roles and hazardous effects of nickel in plants. Rev. Environ. Contam. Toxicol..

[37] Yusuf M., Fariduddin Q., Hayat S., Ahmad A. (2011). Nickel: An overview of uptake, essentiality and toxicity in plants. Bull. Environ. Contam. Toxicol..

[38] Bhalerao S.A., Amit S.S., Anukthi C.P. (2015). Toxicity of nickel in plants. Int. J. Pure Appl. Biosci..

[39] Seregin I.V., Kozhevnikova A.D. (2006). Physiological role of nickel and its toxic effects on higher plants. Russ. J. Plant Physiol..

[40] Wang H.H., Kang J., Zeng F.H., Jiang M.Y. (2001). Effect of nickel at high concentrations on growth activities of enzymes of rice seedlings. Acta Agron. Sin..

[41] Prasad S.M., Dwivedi R., Zeeshan M. (2005). Growth, photosynthetic electron transport, and antioxidant responses of young soybean seedlings to simultaneous exposure of nickel and UV-B stress. Photosynthetica.

[42] Pandey N., Pathak G. (2006). Nickel alters antioxidative defense and water status in green gram. Ind. J. Plant Physiol..

[43] Gajewska E., Skłodowska M. (2007). Effect of nickel on ROS content and antioxidative enzyme activities in wheat leaves. Biometals.

[44] Yan R., Gao S., Yang W., Cao M., Wang S., Chen F. (2008). Nickel toxicity induced antioxidant enzyme and phenylalanine ammonia-lyase activities in *Jatropha curcas* L. cotyledons. Plant Soil Environ..

[45] Mandon K., Pauly N., Boscari A., Brouquisse R., Frendo P., Demple B., Puppo A. (2009). ROS in the Legume-Rhizobium Symbiosis. Reactive Oxygen Species in Plant Signaling.

[46] Foyer C.H. (2018). Reactive oxygen species, oxidative signaling and the regulation of photosynthesis. Environ. Exp. Bot..

[47] Khorobrykh S., Havurinne V., Mattila H., Tyystjärvi E. (2020). Oxygen and ROS in photosynthesis. Plants.

[48] Wituszyńska W., Karpiński S. (2018). Friend or foe? Reactive oxygen species production, scavenging and signaling in plant response to environmental stresses. Free Rad. Biol. Med..

[49] Baker A.J.M., Walker P.L., Shaw A.J. (1990). Ecophysiology of Metal Uptake by Tolerant Plants: Heavy Metal Tolerance in Plants. Evolutionary Aspects.

[50] Duman F., Ozturk F. (2010). Nickel accumulation and its effect on biomass, protein content and antioxidative enzymes in roots and leaves of watercress (*Nasturtium officinale* R. Br.). J. Environ. Sci..

[51] Fukumoto L.R., Mazza G. (2000). Assessing antioxidant and prooxidant activities of phenolic compounds. J. Agric. Food Chem..

[52] Mishra J., Singh R., Arora N.K. (2017). Alleviation of heavy metal stress in plants and remediation of soil by rhizosphere microorganisms. Front. Microbiol..

[53] Żołnierz L. (2007). Grass communities occurring in Lower Silesian serpentine-selected aspects of ecology. Zesz. Nauk. UP Wroc..

[54] Banasiewicz J., Granada C.E., Lisboa B.B., Grzesiuk M., Matuśkiewicz W., Bałka M., Schlindwein G., Vargas L.K., Passaglia L.M.P., Stępkowski T. (2021). Diversity and phylogenetic affinities of *Bradyrhizobium* isolates from pampa and atlantic forest biomes. Syst. Appl. Microbiol..

[55] Zhao C.Z., Huang J., Gyaneshwar P., Zhao D. (2018). *Rhizobium* sp. IRBG74 alters Arabidopsis root development by affecting auxin signaling. Front. Microbiol..

[56] Bai Y., Müller D.B., Srinivas G., Garrido-Oter R., Potthoff E., Rott M., Dombrowski N., Münch P.C., Spaepen S., Remus-Emsermann M. (2015). Functional overlap of the Arabidopsis leaf and root microbiota. Nature.

[57] Mahieu S., Frérot H., Vidal C., Galiana A., Heulin-Gotty K., Maure L., Brunel B., Lefèbvre C., Escarré J., Cleyet-Marel J.-C. (2011). Anthyllis vulneraria/Mesorhizobium metallidurans, an efficient symbiotic nitrogen fixing association able to grow in mine tailings highly contaminated by Zn, Pb and Cd. Plant Soil.

[58] Grison C.M., Mazel M., Sellini A., Escande V., Biton J., Grison C. (2015). The leguminous species *Anthyllis vulneraria* as a Zn-hyperaccumulator and eco-Zn catalyst resources. Environ. Sci. Pollut. Res..

[59] Chaintreuil C., Rigault F., Moulin L., Jaffre T., Fardoux J., Giraud E., Dreyfus B., Bailly X. (2007). Nickel resistance determinants in *Bradyrhizobium* strains from nodules of the endemic New Caledonia legume *Serianthes calycina*. Appl. Environ. Microbiol..

[60] Mengoni A., Schat H., Vangronsveld J. (2010). Plants as extreme environments? Ni-resistant bacteria and Ni-hyperaccumulators of serpentine flora. Plant Soil.

[61] Mergeay M., Monchy S., Vallaeys T., Auquier V., Benotmane A., Bertin F., Taghavi S., Dunn J., van der Lelie D., Wattiez R. (2003). *Ralstonia metallidurans*, a bacterium specifically adapted to toxic metals: Towards a catalogue of metal-responsive genes. FEMS Microbiol. Rev..

[62] Anjum M.A., Zahir Z., Arshad M., Ashraf M. (2011). Isolation and screening of rhizobia for auxin biosynthesis and growth promotion of mung bean (*Vigna radiata* L.) seedlings under axenic conditions. Soil Environ..

[63] Friml J. (2003). Auxin transport-shaping the plant. Curr. Opin. Plant Biol..

[64] Evans M.L., Ishikawa H., Estelle M.A. (1994). Responses of Arabidopsis roots to auxin studied with high temporal resolution: Comparison of wild type and auxin-response mutants. Planta.

[65] Reed R.C., Brady S.R., Muday G.K. (1998). Inhibition of auxin movement from the shoot into the root inhibits lateral root development in Arabidopsis. Plant Physiol..

[66] Ahemad M., Kibret M. (2014). Mechanisms and applications of plant growth-promoting rhizobacteria: Current perspective. J. King Saud Univ. Sci..

[67] Wang F., Cui X., Sun Y., Dong C.H. (2013). Ethylene signaling and regulation in plant growth and stress responses. Plant Cell Rep..

[68] Li J., Ovakim D.H., Charles T.C., Glick B.R. (2000). An ACC deaminase minus mutant of *Enterobacter cloacae* UW4 no longer promotes root elongation. Curr. Microbiol..

[69] Oldroyd G.E.D., Engstrom E.M., Long S.R. (2001). Ethylene inhibits the Nod factor signal transduction pathway of *Medicago truncatula*. Plant Cell.

[70] Kumari P., Meena M., Upadhyay R.S. (2018). Characterization of plant growth promoting rhizobacteria (PGPR) isolated from the rhizosphere of *Vigna radiata* (mung bean). Biocatal. Agric. Biotechnol..

[71] Kumar A., Bahadur I., Maurya B.R., Raghuwanshi R., Meena V.S., Singh D.K., Dixit J. (2015). Does a plant growth promoting rhizobacteria enhance agricultural sustainability?. J. Pure App. Microbiol..

[72] Chi F., Shen S.H., Cheng H.P., Jing Y.X., Yanni Y.G., Dazzo F.B. (2005). Ascending migration of endophytic rhizobia, from roots to leaves, inside rice plants and assessment of benefits to rice growth physiology. Appl. Environ. Microbiol..

[73] Salazear-Cerezo S., Martinez-Montiel N., Garcia-Sanchez J., Perezy-Terron R., Martinez-Contreras D. (2018). Gibberellin biosynthesis and metabolism: A convergent route for plants, fungi and bacteria. Microbiol. Res..

[74] Kalaji H.M., Jajoo A., Oukarroum A., Brestic M., Zivcak M., Samborska I.A., Cetner M.D., Łukasik I., Goltsev V., Ladle R.J. (2016). Chlorophyll a fluorescence as a tool to monitor physiological status of plants under abiotic stress conditions. Acta Physiol. Plant.

[75] Arumugam R., Rajasekaran S., Nagarajan S.M. (2010). Response of arbuscular mycorrhizal fungi and Rhizobium inoculation on growth and chlorophyll content of *Vigna unguiculata* (L.) Walp Var. Pusa 151. J. Appl. Sci. Environ..

[76] Yadav A., Yadav K., Tanwar A., Aggarwal A. (2013). Interaction of VAM Fungi with *Bradyrhizobium japonicum* and *Trichoderma viride* on some physiological parameters of soybean. J. Pure Appl. Microbiol..

[77] Ainsworth E.A., Bush D.R. (2011). Carbohydrate export from the leaf: A highly regulated process and target to enhance photosynthesis and productivity. Plant Physiol..

[78] Kalaji H.M., Pathom-Aree W., Lotfi R., Balaji P., Elshery N., Grska E.B., Swiatek M., Horaczek T., Mojski J., Kociel H. (2018). Effect of microbial consortia on photosynthetic efficiency of *Arabidopsis thaliana* under drought stress. Chiang Mai J. Sci..

[79] Puppo A., Pauly N., Boscari A., Mandon K., Brouquisse R. (2013). Hydrogen peroxide and nitric oxide: Key regulators of the *Legume-Rhizobium* and mycorrhizal symbioses. Antioxid. Redox Signal..

[80] Stearns J.C., Shah S., Greenberg B.M., Dixon D.G., Glick B.R. (2005). Tolerance of transgenic canola expressing 1-aminocyclopropane-1-carboxylic acid deaminase to growth inhibition by nickel. Plant Physiol. Biochem..

[81] Barnawal D., Bharti N., Pandey S.S., Pandey A., Chanotiya C.S., Kalra A. (2017). Plant growth-promoting rhizobacteria enhance wheat salt and drought stress tolerance by altering endogenous phytohormone levels and TaCTR1/TaDREB2 expression. Physiol. Plant..

[82] Molas J. (2002). Changes of chloroplasts ultrastructure and total chlorophyll concentration in cabbage leaves caused by excess of organic Ni(II) complexes. Environ. Exp. Bot..

[83] Wituszyńska W., Gałązka K., Rusaczonek A., Vanderauwera S., Van Breusegem F., Karpiński S. (2015). Multivariable environmental conditions promote photosynthetic adaptation potential in *Arabidopsis thaliana*. J. Plant Physiol..

[84] Hao F.S., Wang X.C., Chen J. (2006). Involvement of plasma-membrane NADPH oxidase in nickel-induced oxidative stress in roots of wheat seedlings. Plant Sci..

[85] Mesa-Marín J., Pérez-Romero J.A., Redondo-Gómez S., Pajuelo E., Rodríguez-Llorente I.D., Mateos-Naranjo E. (2020). Impact of plant growth promoting bacteria on Salicornia ramosissima ecophysiology and heavy metal phytoremediation capacity in estuarine soils. Front. Microbiol..

[86] Shahid M., Pourrut B., Dumat C., Nadeem M., Aslam M., Pinelli E. (2014). Heavy-metal-induced reactive oxygen species: Phytotoxicity and physicochemical changes in plants. Rev. Environ. Contam. Toxicol..

[87] Ju W., Liu L., Fang L., Cui Y., Duan C., Wu H. (2019). Impact of co-inoculation with plant-growth-promoting rhizobacteria and rhizobium on the biochemical responses of alfalfa-soil system in copper contaminated soil. Ecotoxicol. Environ. Saf..

[88] Aqeel M., Khalid N., Tufail A., Ahmad R.Z., Akhter M.S., Luqman M., Javed M.T., Irshad M.K., Alamri S., Hashem M. (2021). Elucidating the distinct interactive impact of cadmium and nickel on growth, photosynthesis, metal-homeostasis, and yield responses of mung bean (*Vigna radiata* L.) varieties. Environ. Sci. Pollut. Res. Int..

[89] Vincent J.M. (1970). A Manual for the Practical Study of Root Nodule Bacteria.

[90] Versalovic J., Schneider M., de Bruijn F.J., Lupski J.R. (1994). Genomic fingerprinting of bacteria with repetitive sequence-based polymerase chain reaction. Methods Mol. Cell Biol..

[91] Debojyoti R., Manibrata P., Sudip K.B. (2015). Isolation identification and characterization of bacteria (*Rhizobium*) from chick pea (*Cicer arietinum*) and production of biofertilizer. Eur. J. Biotech. Biosci..

[92] Aryantha I.N.P., Hidiyah A.R.M. (2018). Colonization and performance of diazotroph endophytic bacteria on palm oil (*Elaeis guineensis* Jacq L.) leaves. IOP Conf. Ser. Earth Environ. Sci..

[93] Goldstein A.H. (1995). Recent progress in understanding the molecular genetics and biochemistry of calcium phosphate solubilization by gram negative bacteria. Biol. Agric. Hortic..

[94] Gordon S.A., Weber R.P. (1951). Colorimetric estimation of indole acetic acid. Plant Physiol..

[95] Penrose D.M., Glick B.R. (2003). Methods for isolating and characterizing ACC deaminase-containing plant growth-promoting rhizobacteria. Physiol. Plant..

[96] Rusaczonek A., Czarnocka W., Kacprzak S., Witoń D., Ślesak I., Szechyńska-Hebda M., Gawroński P., Karpiński S. (2015). Role of phytochromes A and B in the regulation of cell death and acclimatory responses to UV stress in *Arabidopsis thaliana*. J. Exp. Bot..

[97] Mergemann H., Sauter M. (2000). Ethylene induces epidermal cell death at the site of adventitious root emergence in rice. Plant Physiol..

[98] Ainsworth E.A., Gillespie K.M. (2007). Estimation of total phenolic content and other oxidation substrates in plant tissues using Folin-Ciocalteu reagent. Nat. Protoc..

[99] Lepš J., Šmilauer P. (2003). Multivariate Analysis of Ecological Data Using Canoco.

[100] Ter Braak C.J.F., Šmilauer P. (2002). CANOCO Reference Manual and CanoDraw for Windows User’s Guide: Software for Canonical Community Ordination (version 4.5). Microcomput. Power Ithaca.

