# Supplementary material for: Potential of Rhizobia Nodulating Anthyllis vulneraria L. from Ultramafic Soil as Plant Growth Promoting Bacteria Alleviating Nickel Stress in Arabidopsis thaliana L
Source: Int J Mol Sci. 2022 Sep 29;23(19):11538. doi: 10.3390/ijms231911538 (PMC9570232; doi:10.3390/ijms231911538)
Supplement: Supplementary file 1 [file ijms-23-11538-s001.zip › ijms-1876381-supplementary.pdf]

Supplementary Materials:

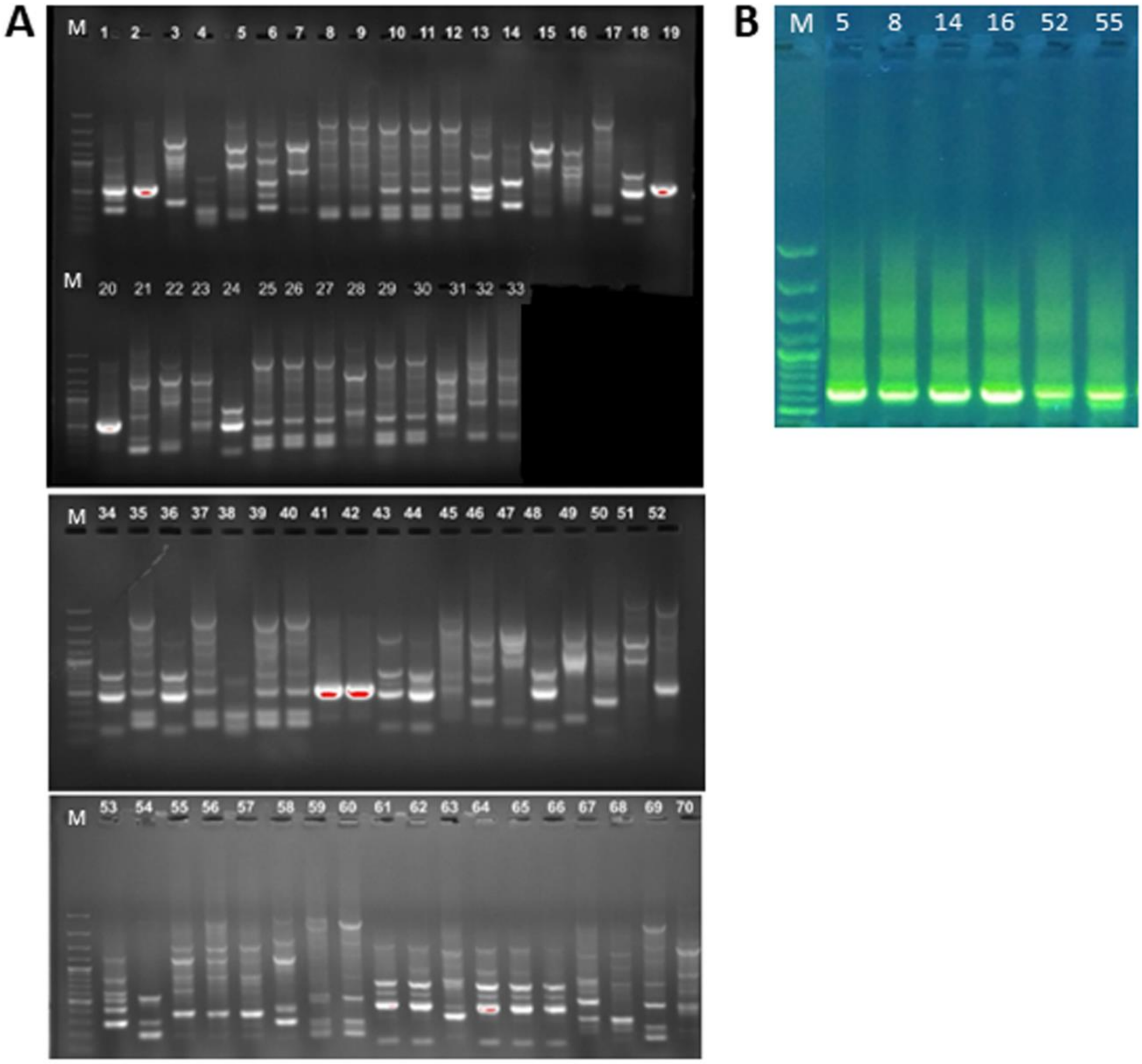

Figure S1. (A) BOX-PCR profiles of *Anthyllis vulneraria* rhizobial isolates from ultramafic soil. (B) PCR amplification of the *glnII*. The numbers on the lanes are the isolate designations. The M lanes are DNA ladder that has bands of known sizes in base pairs (bp).

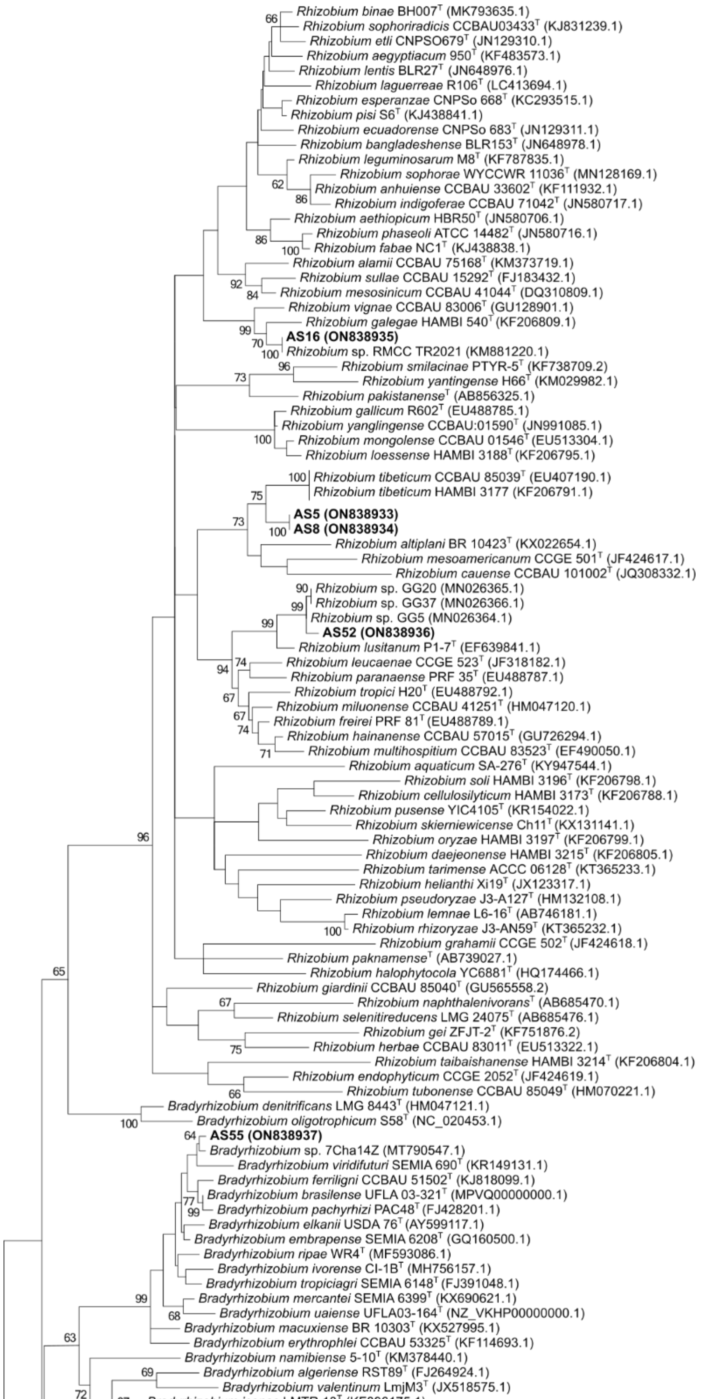

Figure S2. Maximum likelihood (ML) tree based on partial *glnII* gene sequences (470 bp), comprising type strains of *Bradyrhizobium* and *Rhizobium* species and the most similar strains. Node robustness was evaluated by using 500 bootstrap replications. Bootstrap values ≥ 60% are shown at each node. The total number of sequences used in the construction of this phylogenetic tree is 139 and the sequence of *Methylobacterium nodulans* ORS 2060 was used as an outgroup. Our AS isolates are marked in bold.

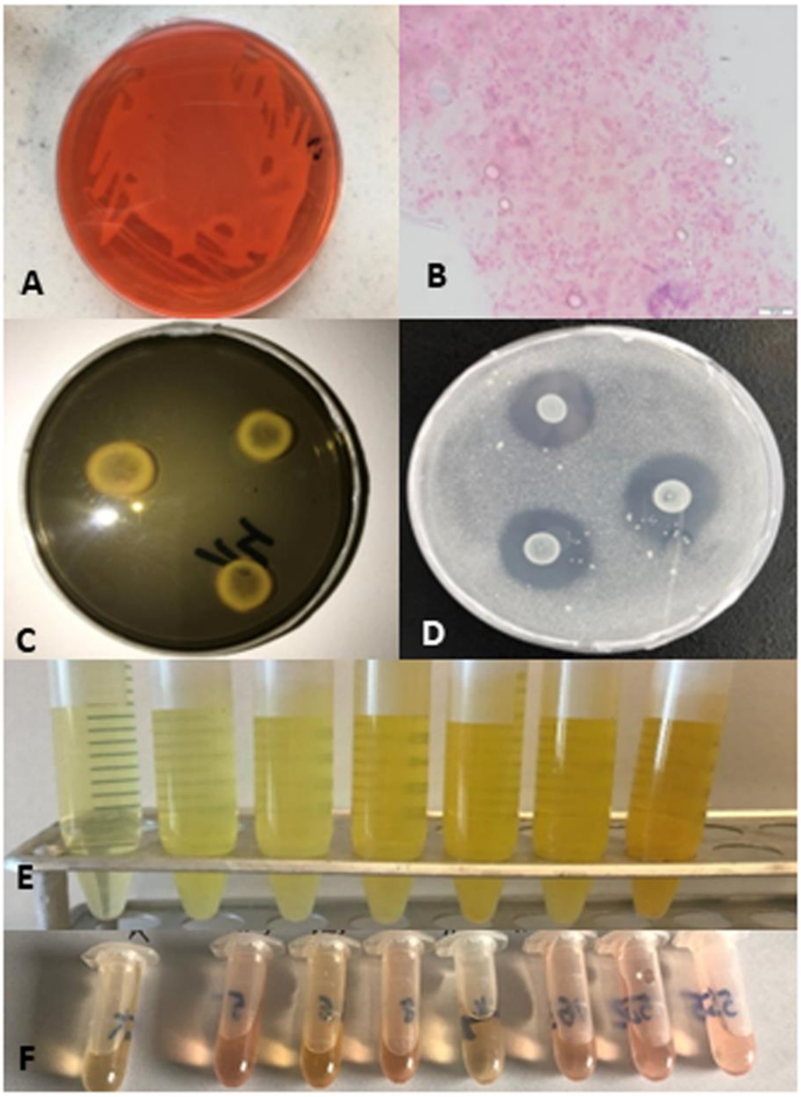

Figure S3. Characteristics of ultramafic *Anthyllis vulneraria* nodulating bacteria and their *in vitro* plant growth promoting traits. (A) Purified cultures on Yeast Extract Congo Red agar media. (B) Gram staining. (C) Orange halo zone on CAS agar plates indicates siderophore production. (D) Isolates showing halo zones indicated phosphate solubilization. (E) Ammonia production. (F) Development of pink color indicated IAA production.

Table S1. Primers used for PCR and qPCR in Arabidopsis

	**Accession number**	**Forward**	**Reverse**
GA20ox3	AT5G07200	CAACCTCTCCAAGTCCCACTC	TGTTTCGTTGCAGCCTTTGAG
GA3ox	At1g80340	CCAGCCACCACCTCAAATACT	ACTCCCAGTGAACCTAATGCG
YUC2	AT4G13260	CCTTGAGTCTTACGCCGAACA	CTCACCCTCCATAACCCACAC
IAA19	AT3G15540	GGGAGAGATGTGGCAGAGAAG	GCCGCTTTCACATTGATCACT
